# Antibacterial and Antitumor Activities of Synthesized Sarumine Derivatives

**DOI:** 10.3390/ijms252212412

**Published:** 2024-11-19

**Authors:** Fangzhou Yang, Bin Jia, Hongli Wen, Xiufang Yang, Yangmin Ma

**Affiliations:** 1 Key Laboratory of Chemical Additives for China National Light Industry, College of Chemistry and Chemical Engineering, Shaanxi University of Science & Technology, Xi’an 710021, China; fangzyang@polyu.edu.hk (F.Y.); jiabin@sust.edu.cn (B.J.); whl1234567891106@163.com (H.W.); yangxf@sust.edu.cn (X.Y.); 2 Department of Applied Biology and Chemical Technology and Research Institute for Smart Energy, The Hong Kong Polytechnic University, Hung Hom, Hong Kong, China

**Keywords:** sarumine, antibacterial activity, total synthesis, molecular dock

## Abstract

Our aim in this study was to explain the biological activity of the latest azafluoranthene. The natural product sarumine (**12**) and its derivatives (**13**–**17**) were synthesized and evaluated for their antibacterial and antitumor activities. The synthesis involved a simplified reaction pathway based on biaryl-sulfonamide-protected cyclization, and the compounds were characterized and studied using spectroscopic methods (^1^HNMR and ^13^CNMR). Most of the compounds demonstrated improved antibacterial activity. Notably, sarumine demonstrated potent activity against *S. aureus* and *B*. *subtilis*, with an MIC of 8 μg/mL, showing comparable inhibitory effects to the positive control. Furthermore, molecular simulation studies indicated that sarumine exhibited significant binding affinity to FabH. The inhibitory effect of Cl was superior to the others on the structure, and the antitumor activity result also suggested that the inhibitory ability in PC-3 displayed by the R_1_ derivatives of F and Cl substitutions was better than that of MDA-MB-231. These findings suggest that sarumine and its derivatives may represent new and promising candidates for further study.

## 1. Introduction

**Figure 1:**
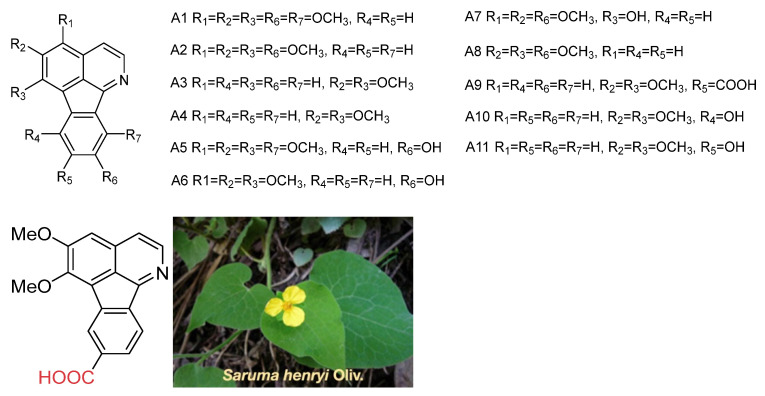
The structure of sarumine (**12**). Red mark: The only natural product confirmed to have a carboxyl group in azafluoranthene.

Sarumine is an innovative azafluoranthene alkaloid delivering advanced antibacterial activity and inhibitory effects. As a niche class of natural occurring alkaloids, only 11 azafluoranthene alkaloids has been reported so far: imeluteine (A1) [1], rufescine (A2) [2], triclisine (A3) [3], telitoxine (A4) [4], norimeluteine (A5) [4], norrufescine (A6) [5], 4,5,9-trimethoxy-indenol [1,2,3-ij]isoquinolin-6-ol (A7) [4], 5,6,9-trimethoxy-indenol [1,2,3-ij] isoquinolin (A8) [6], sarumine (A9) [1], tatarine A (A10) [7], and tatarine D (A11) [7], as shown in Figure 1. Most of them have been isolated from plants in the Neisseriaceae family. Azafluoranthene alkaloids have displayed interesting biological activities. Preliminary studies on the biological activity of these compounds revealed that norimeluteine and norrufescine display significant antitumor activity against P-388 cells at IC_50_ values of 3.6 and 5 against human immunodeficiency virus (HIV-1), with EC_50_ values of 10.9 μg/mL and 6 μg/mL [6,8,9,10,11]. Norrufescine was also reported to exhibit inhibitory activity against A549 lung carcinoma with an IC_50_ value of 31.0 μg/mL [7,11]. Furthermore, the cytotoxic activity of the azafluoranthene eupolauridine was attributed to the targeting of DNA topoisomerase II [10,12]. Additionally, regarding biological activities, the extensive conjugation in molecules containing an azafluoranthene core endows them with interesting spectral properties [13]. Thus, they have been studied as ideal lead compounds for the development of drugs to combat diseases and as potentially useful agents in luminescence. It is important to devise a synthesis approach for increasing the class of azafluoranthene alkaloids and for studying structure–activity relationships.

Several strategies have been employed to synthesize azafluoranthene alkaloids, which include oxidative biaryl cyclization [14], photocyclization [15], electrocyclization mediated approach [16], combined aza-Wittig/electrocyclic ring closure, and biaryl-sulfonamide-protected cyclization [10,17,18]. However, these routes have the drawbacks of low yields as well as requiring long synthesis sequences. To overcome the weakness of the general synthesis routes, we not only introduced an improved synthesis method that simplifies the reaction steps based on biaryl-sulfonamide-protected cyclization and the antibacterial activity of sarumine derivatives but also studied the mechanism of protein inhibition and the binding mode of the compounds via molecular simulation.

## 2. Results and Discussion

### 2.1. Synthesis

**Scheme 1:**
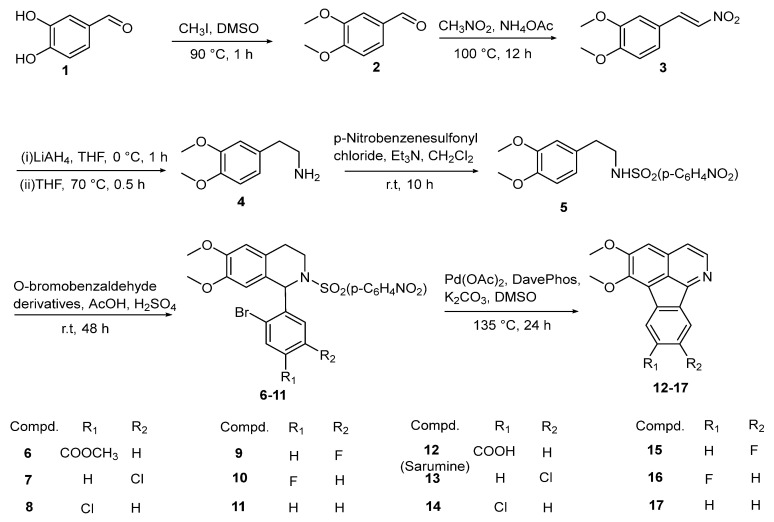
Synthesis route of compounds **12**–**17**.

Compounds **2**–**17** were prepared as described in Scheme 1, involving the synthesis of a series of sarumine derivatives starting from commercially available 3,4-dihydroxybenzaldehyde 1. Initially, compound **1** underwent nucleophilic substitution with iodomethane in DMSO at 90 °C for 1 h to yield compound **2**, which was subsequently purified by column chromatography on silica gel (94% yield). Subsequently, compound **2** was subjected to a reaction with CH_3_NO_2_ in acetic acid, using NH_4_OAc as a catalyst, then refluxed at 100 °C for 12 h. Nitromethane was employed as the nucleophilic reagent to react with compound **2**, resulting in the elimination of nucleophilic addition and yielding compound **3**, which was purified by recrystallization (93% yield). Compound **3** was then treated with LiAlH_4_ in dry THF at a reflux temperature of 70 °C for 0.5 h to reduce the nitro group to an amino group. The reaction mixture was subsequently poured into ice water, followed by the addition of NaOH to adjust the pH, and the resulting mixture was filtered to obtain compound **4** (89% yield). Compound **4** was reacted with p-nitrobenzene sulfonyl chloride in CH_2_Cl_2_ at room temperature to obtain compound **5** without any purification (95% yield). Compound **5** underwent a reaction with o-bromobenzaldehyde derivatives at room temperature for 48 h under the catalysis of H_2_SO_4_ in acetic acid. The reaction mechanism involved the nucleophilic addition of the aldehyde group and imino group to form an intermediate, followed by nucleophilic substitution with hydrogen on the aromatic ring to yield compounds **6**–**11** (80–92% yield) without any purification. Finally, compounds **6**–**11** were prepared using Pd(OAc)_2_ as a catalyst, as well as DavePhos and K_2_CO_3_ as additives in DMSO, and refluxed at 135 °C for 12 h to obtain compounds **12**–**17** (31–71% yield). In comparison with the general synthesis route, the coupling, elimination, and oxidation reactions occurred simultaneously in one step in this method. The detailed spectral data of compounds **2**–**17** are listed in the Supporting Information.

### 2.2. Screening Reaction Conditions

The synthesis of a natural product, sarumine, is summarized in Table 1. A one-pot synthesis of sarumine was conducted to improve the product yield by optimizing the reaction conditions. Compound **6** was used as the substrate, with Pd(OAc)_2_ as the catalyst and DMSO as the solvent. Ligands were screened at 100 °C, and DavePhos exhibited the highest yield at 55%. Subsequently, solvent screening revealed the highest yield in the polar solvent DMSO at 59%, followed by DMF, while the low-polarity solvent dichloromethane did not yield any product. Finally, temperature screening showed the highest yield at 71% at 135 °C.

### 2.3. Antibacterial Activities of Compounds **12**–**17**

As reported in Table 2, compounds **12**–**17** were tested for their antibacterial activity against *S. aureus, B. subtilis, P. aeruginosa,* and *E. coli.*

In this series, two compounds (**13**, **17**) exhibited poor activity against all bacteria. Compound **17**, lacking a substituent on the aromatic ring, demonstrated weak activity against *S. aureus* and *B. subtilis* with an MIC of 256 μg/mL and against *P. aeruginosa* and *E. coli* with an MIC of 128 μg/mL, as indicated in Table 2. Conversely, compounds **13** and **14,** featuring a Cl on the aromatic ring, displayed limited antibacterial activity, with MICs of 64~128 μg/mL against the four bacteria. Meanwhile, compounds **15** and **16**, containing a F on the aromatic ring, demonstrated moderate activity against *P. aeruginosa* and *E. coli* with MICs of 64 and 32 μg/mL, respectively. Both **15** and **16** exhibited the same MIC value against *S. aureus* and *B. subtilis* at 64 μg/mL. Notably, sarumine, with a carboxyl group on the aromatic ring, exhibited potent activity against *S. aureus* and *B. subtilis* with an MIC of 8 μg/mL and against *P. aeruginosa* and *E. coli* with an MIC 16 of μg/mL, showing similar inhibitory effects to the positive control. This confirmed the antibacterial activity was improved with a carboxyl group on the aromatic ring, which was crucial for modifying the enzymatic activity. Additionally, the inhibitory effect of F was superior to that of Cl on the structure.

### 2.4. Lipophilicity

Lipophilicity is a crucial parameter for elucidating the mechanism of action of antimicrobial agents against pathogenic bacteria [19]. Consequently, the theoretical partition coefficients (Clog p) were considered in the present analysis. A significant correlation was observed between the antimicrobial activity of the sarumine derivatives and their lipophilicity. Our findings indicate that sarumine and several synthesized molecules exhibited promising antimicrobial activities, accompanied by lower Clog p values, particularly in compounds demonstrating high activity. Sarumine exhibited good lipophilicity (Clog p, 3.71). Although it contains a carboxyl group in its structure, it is lipophilic due to the influence of the aryl isoquinoline parent and the methoxy group. In addition, compounds with stronger hydrophobicity are more likely to enter the protein cavity. Based on the results, it can be inferred that increased hydrophobicity facilitates the uptake of reagents by bacteria, as indicated by lower Clog p values. Typically, Clog p values of about three are deemed optimal for oral drugs [20]. Furthermore, it has been suggested that ligands with a Clog p value of less than five exhibit a more favorable drug-likeness profile [21]. In general, sarumine exhibited moderate Clog P values, suggesting its potential as a drug candidate.

### 2.5. Molecular Docking

**Figure 2:**
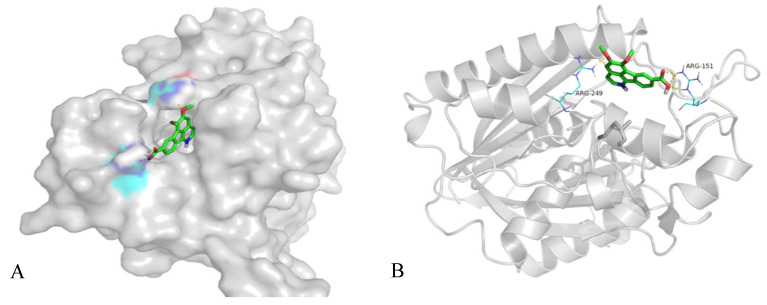
(**A**) Docking simulation of binding mode between sarumine cand FabH. The receptor FabH is shown in surface view, while the structure of sarumine is shown as sticks. (**B**) The 3D diagram of sarumine’s interaction with the FabH active site (colored in green is C, red is O, blue is N). The proteins are shown in cartoon representation with the ligands drawn as sticks.

**Figure 3:**
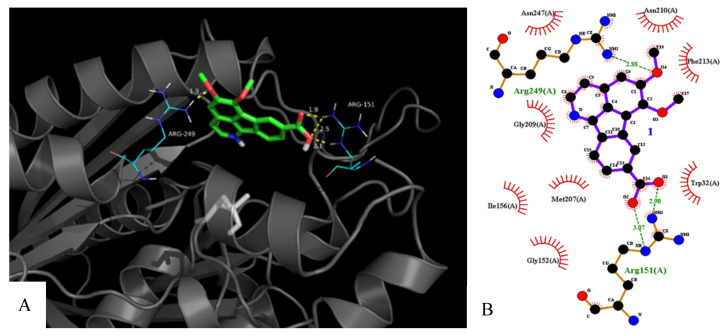
(**A**) Docking of sarumine (colored in green is C, red is O, blue is N) into the active site of FabH (gray, PDB: 1HNJ) via hydrogen bonding with Arg249 and Arg151 (colored in blue). Likely hydrogen bonds are indicated with yellow dashed lines (three hydrogen bonds). (**B**) The details of the interactions were visualized using ligplus v1.4.5.

The enzyme FabH is responsible for the initial stage of synthesis of bacterial fatty acids, the inhibition of which can result in the interference with bacterial cell membrane formation and the development of biofilms [22], consequently inhibiting the growth and reproduction of bacteria [23]. The structures containing methoxy and carboxyl groups were more likely to form hydrogen bonds with FabH to inhibit the activity of the FabH enzyme [24,25]. In addition, the lower the energy required for binding, the stronger the affinity, and the better the antibacterial effect [26]. In this study, we conducted molecular docking studies involving sarumine with the crystal structure of FabH (Protein Data Bank entry 1HNJ) to elucidate the binding mode of sarumine. These molecular docking studies offered valuable insights into the active mechanism of sarumine; the results of the docking studies are presented in Table 3 and Figure 2 and Figure 3. As shown in Table 3, the binding free energy of sarumine (∆G_b_, kcal/mol, −9.885) was the lowest due to the formation of hydrogen bonds between the carboxyl group and the protein cavity, having a lower binding energy than the other derivatives, resulting in the strongest antibacterial activity.

As shown in Figure 2, the FabH binding site encapsulates sarumine in the hydrophobic pocket. Consistent with the docking calculations, sarumine exhibited significant binding affinity to FabH, which forms three hydrogen bonds with the key residues, Arg249 and Arg151, and fits well within the FabH active pocket with a very low interaction energy. The methoxy oxygen of sarumine forms a hydrogen bond with the NH group on Arg249, while the carboxyl oxygen and hydroxyl oxygen of sarumine form hydrogen bonds with the NH group on Arg151, as shown in Figure 3. The formation of these three hydrogen bonds, combined with the interaction with the hydrophobic cavity, contributes to the good antibacterial activity of sarumine.

### 2.6. Antitumor Activities of Compounds **12**–**17**

To explore the anticancer activity of the synthesized compounds, the antitumor activities of sarumine and its derivatives in PC-3 and MDA-MB-231 cells were determined. Screening showed that several sarumine derivatives have antiproliferative activity. As shown in Table 4, with adriamycin as the positive control, the IC_50_ values of the screened compounds on PC-3 and MDA-MB-231 cells were higher than those of cisplatin; compounds **12** (sarumine), **13**, and **14**, exhibited moderate antiproliferative activity against one or more cancer cells. Among them, compound **14** had the strongest antiproliferative activity; its IC_50_ values for the two cancer cell lines were 7 μM and 9 μM, respectively. This confirmed that the inhibitory effect of the Cl was superior to that of the others on the structure, and the result suggested that the inhibitory ability displayed by the derivatives of R_1_ with F and Cl substitutions on PC-3 is better than MDA-MB-231; derivatives of R_2_ with F and Cl substitutions on MDA-MB-231 were stronger than PC-3, indicating that PC-3 is more sensitive to derivatives of R_1_ with F and Cl substitutions than MDA-MB-231, and MDA-MB-231 is more sensitive to derivatives of R_2_ with F and Cl substitutions than PC-3.

## 3. Materials and Methods

### 3.1. Chemistry

All solvents and reagents were utilized as commercially available without further purification. ^1^H NMR and ^13^C NMR spectra were acquired using an AVANCE III 400 MHz spectrometer (Bruker) with tetramethyl silane (TMS) as the internal standard. Chemical shifts are reported in parts per million (ppm, δ scale), and coupling constants (J) are expressed in hertz (Hz). Mass spectra were obtained using a QEXACTIVE mass spectrometer (ThermoFisher Scientific). Thin-layer chromatography (TLC) was conducted on precoated plates (silica gel 300–400 mesh), and compounds were visualized under UV light.

#### 3.1.1. General Procedure for Preparation of Compounds **2**–**5**

N-(3,4-dimethoxyphenethyl)-4-nitrobenzenesulfonamide (**2**–**5**) was prepared and purified following the reported methods [16].

#### 3.1.2. General Procedure for Preparation of Compounds **6**–**11**

A solution of compound **5** (800 mg, 2.18 mmol) and 3-bromo-4-formylbenzoic acid (496 mg, 2.18 mmol) in acetic acid was treated with concentrated sulfuric acid (6 mL) dropwise at 0 °C. The reaction mixture was stirred at room temperature for 24 h, followed by the addition of additional concentrated sulfuric acid (3 mL). After stirring at room temperature for another 24 h, the reaction mixture was poured into ice water (30 mL) and extracted with CH_2_Cl_2_ (20 mL × 3). The organic layer was washed with water, dried with MgSO_4_, and filtered, and the solvent was evaporated under reduced pressure to yield compound **6** as a yellow solid with 84% yield. The resulting crude product was used directly without further purification in the subsequent step. Compounds **7**–**11** were prepared using the same method as described for compound **6**.

Methyl-3-bromo-4-(6,7-dimethoxy-2-((4-nitrophenyl)sulfonyl)-1,2,3,4-tetrahydroisoquinolin-1-yl)benzoate (**6**). Yield 89%; yellow solid; ^1^H NMR (400 MHz, chloroform-d) δ 8.29 (d, J = 1.7 Hz, 1H), 8.25–8.18 (m, 2H), 7.95–7.87 (m, 2H), 7.75 (dd, J = 8.2, 1.7 Hz, 1H), 7.10 (d, J = 8.2 Hz, 1H), 6.56 (s, 1H), 6.48 (d, J = 5.8 Hz, 2H), 3.94 (s, 3H), 3.88 (dd, J = 9.1, 4.3 Hz, 1H), 3.85 (s, 3H), 3.73 (s, 3H), 3.68 (ddd, J = 13.7, 8.2, 5.9 Hz, 1H), 2.89 (t, J = 4.9 Hz, 2H). 13C NMR (101 MHz, CDCl3) δ 165.13, 149.89, 148.71, 148.36, 146.26, 145.18, 134.45, 131.19, 130.34, 128.56, 128.50, 125.55, 125.07, 123.91, 123.69, 111.30, 109.84, 58.27, 55.92, 52.49, 41.61, 27.72.

1-(2-bromo-5-chlorophenyl)-6,7-dimethoxy-2-((4-nitrophenyl)sulfonyl)-1,2,3,4-tetrahydroisoquinoline (**7**). Yield 87%; yellow solid; ^1^H NMR (400 MHz, chloroform-d) δ 8.22 (d, J = 8.5 Hz, 2H), 7.88 (d, J = 8.4 Hz, 2H), 7.57 (d, J = 8.5 Hz, 1H), 7.10 (dd, J = 8.6, 2.4 Hz, 1H), 6.90 (d, J = 2.4 Hz, 1H), 6.58 (s, 1H), 6.47 (d, J = 24.8 Hz, 2H), 3.95 (ddd, J = 15.2, 11.7, 4.9 Hz, 2H), 3.87 (s, 3H), 3.76 (s, 3H), 3.67 (dt, J = 13.9, 7.2 Hz, 1H), 2.90 (t, J = 5.9 Hz, 2H). ^13^C NMR (101 MHz, chloroform-d) δ 149.85, 148.72, 148.35, 145.43, 143.18, 134.41, 133.75, 130.27, 129.52, 128.26, 125.44, 125.06, 123.87, 121.72, 111.32, 109.88, 58.26, 55.93, 41.63, 27.83.

1-(2-bromo-4-chlorophenyl)-6,7-dimethoxy-2-((4-nitrophenyl)sulfonyl)-1,2,3,4-tetrahydroisoquinoline (**8**). Yield 85%; yellow solid; ^1^H NMR (400 MHz, chloroform-d) δ 8.24 (d, J = 8.4 Hz, 2H), 7.93 (d, J = 8.4 Hz, 2H), 7.66 (s, 1H), 7.32 (s, 1H), 7.13 (d, J = 8.4 Hz, 1H), 6.93 (d, J = 8.5 Hz, 1H), 6.55 (s, 1H), 6.46 (d, J = 12.1 Hz, 2H), 3.87 (s, 5H), 3.78 (s, 3H), 3.61 (dt, J = 14.0, 7.2 Hz, 1H), 2.85 (q, J = 5.7, 3.7 Hz, 2H), 0.06 (d, J = 2.4 Hz, 1H). ^13^C NMR (101 MHz, chloroform-d) δ 148.78, 148.38, 139.94, 134.63, 132.97, 131.44, 128.51, 127.90, 125.75, 125.11, 124.26, 123.87, 111.29, 109.98, 58.08, 55.98, 55.94, 41.05, 27.39.

1-(2-bromo-5-fluorophenyl)-6,7-dimethoxy-2-((4-nitrophenyl)sulfonyl)-1,2,3,4-tetrahydroisoquinoline (**9**). Yield 92%; yellow solid; ^1^H NMR (400 MHz, chloroform-d) δ 8.25 (dd, J = 8.8, 2.1 Hz, 2H), 7.92 (dd, J = 8.8, 2.1 Hz, 2H), 7.82 (d, J = 2.4 Hz, 1H), 7.27 (d, J = 8.5 Hz, 1H), 6.87 (dd, J = 8.4, 2.4 Hz, 1H), 6.55 (s, 1H), 6.51–6.41 (m, 2H), 3.87 (d, J = 2.2 Hz, 4H), 3.78 (d, J = 2.1 Hz, 3H), 3.62 (dt, J = 13.7, 7.2 Hz, 1H), 2.86 (q, J = 5.6, 4.5 Hz, 2H). ^13^C NMR (101 MHz, chloroform-d) δ 149.87, 148.77, 148.37, 145.49, 140.42, 135.68, 131.74, 130.81, 128.48, 125.66, 125.08, 124.53, 123.86, 122.45, 111.27, 109.94, 58.13, 55.97, 55.92, 41.09, 27.42.

1-(2-bromo-4-fluorophenyl)-6,7-dimethoxy-2-((4-nitrophenyl)sulfonyl)-1,2,3,4-tetrahydroisoquinoline (**10**). Yield 91%; yellow solid; ^1^H NMR (400 MHz, chloroform-d) δ 8.21 (dd, J = 8.7, 1.7 Hz, 2H), 7.90 (dd, J = 8.7, 1.7 Hz, 2H), 7.37 (dt, J = 8.3, 2.0 Hz, 1H), 7.29 (s, 1H), 6.95 (t, J = 7.4 Hz, 1H), 6.92–6.82 (m, 1H), 6.51 (s, 1H), 6.44 (s, 2H), 3.84 (d, J = 1.5 Hz, 4H), 3.75 (d, J = 1.6 Hz, 3H), 3.63–3.50 (m, 1H), 2.81 (dt, J = 9.1, 4.9 Hz, 2H). ^13^C NMR (101 MHz, chloroform-d) δ 149.86, 148.73, 148.32, 145.66, 137.21, 131.87, 131.78, 128.51, 125.97, 125.08, 123.82, 120.68, 120.43, 114.95, 114.74, 111.21, 109.98, 58.00, 55.96, 55.92, 40.76, 27.21.

1-(2-bromophenyl)-6,7-dimethoxy-2-((4-nitrophenyl)sulfonyl)-1,2,3,4-tetrahydroisoquinoline (**11**). Yield 88%; yellow solid; ^1^H NMR (400 MHz, chloroform-d) δ 8.22–8.15 (m, 2H), 7.91–7.84 (m, 2H), 7.66–7.59 (m, 1H), 7.18–7.06 (m, 2H), 6.98 (dd, J = 7.1, 2.3 Hz, 1H), 6.58–6.46 (m, 3H), 3.97 (d, J = 12.6 Hz, 1H), 3.86 (s, 3H), 3.75 (s, 2H), 3.63 (ddd, J = 14.1, 10.1, 5.0 Hz, 1H), 2.87 (dtdd, J = 30.9, 18.9, 11.8, 6.3 Hz, 2H). ^13^C NMR (101 MHz, chloroform-d) δ 149.73, 148.57, 148.25, 145.83, 141.13, 133.45, 130.65, 129.45, 128.40, 127.60, 126.25, 125.11, 124.08, 123.75, 111.19, 110.12, 58.42, 55.94, 41.08, 27.70.

#### 3.1.3. General Procedure for Preparation of Compounds **12**–**17**

Compound **6** (125 mg, 0.2 mmol), Pd(OAc)_2_ (4.8 mg, 0.02 mmol), ligand (16 mg, 0.04 mmol), and K_2_CO_3_ (95 mg, 0.8 mmol) were combined and dissolved in anhydrous purged Ar, degassed DMSO (1 mL) in a sealed tube. The reaction mixture was stirred at 135 °C for 24 h. After cooling to room temperature, the reaction mixture was quenched with 10 wt.% HCl (20 mL) and extracted with ethyl acetate (20 mL × 3). The filtrate was dried over MgSO_4_ and filtered, and the products were concentrated under reduced pressure to yield a brown liquid. The resulting yellow solid was recrystallized by adding ethyl acetate and n-hexane. The crude product was then purified by flash column chromatography (EtOAc/hexanes = 1:1) to afford compound **12** with a 50% yield. Compounds **13**–**17** were prepared using the same method as applied to produce compound **12** (sarumine).

5,6-dimethoxyindeno [1,2,3-ij]isoquinoline-8-carboxylic acid (**12**). Yield 50%; yellow solid; ^1^H NMR (400 MHz, DMSO-d6) δ 8.65 (d, J = 5.6 Hz, 1H), 8.60 (s, 1H), 8.18 (d, J = 7.9 Hz, 1H), 8.06 (d, J = 7.8 Hz, 1H), 7.72 (d, J = 5.6 Hz, 1H), 7.40 (s, 1H), 4.11 (s, 3H), 3.96 (s, 3H). ^13^C NMR (101 MHz, DMSO-d) δ 166.76, 155.98, 154.90, 149.28, 144.69, 142.00, 138.44, 130.42, 128.68, 128.52, 124.62, 124.24, 123.41, 121.66, 118.53, 105.39, 57.26, 52.72. HRMS (ESI) m/z calcd for C_18_H_13_NO_4_ [M + H]^+^: 308.0923, found 308.0917.

9-chloro-5,6-dimethoxyindeno [1,2,3-ij]isoquinoline (**13**). Yield 31%; yellow solid; ^1^H NMR (400 MHz, chloroform-d) δ 8.63 (d, J = 5.9 Hz, 1H), 8.20 (s, 1H), 7.98 (d, J = 7.9 Hz, 1H), 7.54 (d, J = 5.8 Hz, 1H), 7.47 (d, J = 8.1 Hz, 1H), 7.08 (d, J = 2.7 Hz, 1H), 4.17 (d, J = 2.6 Hz, 3H), 4.11 (d, J = 2.5 Hz, 3H). 13C NMR (101 MHz, chloroform-d) δ 159.41, 147.93, 138.18, 131.00, 130.11, 128.59, 126.61, 124.87, 123.46, 122.40, 117.31, 104.82, 77.33, 61.51, 56.53. HRMS (ESI) m/z calcd for C_17_H_13_ClNO_2_ [M + H]^+^: 298.0635, found 298.0629.

8-chloro-5,6-dimethoxyindeno [1,2,3-ij]isoquinoline (**14**). Yield 34%; yellow solid; ^1^H NMR (400 MHz, chloroform-d) δ 8.54 (d, J = 5.4 Hz, 1H), 8.08 (s, 1H), 7.89 (d, J = 7.6 Hz, 1H), 7.44 (d, J = 5.3 Hz, 1H), 7.38 (d, J = 8.0 Hz, 1H), 7.24 (s, 2H), 6.99 (d, J = 3.6 Hz, 1H), 4.09 (d, J = 3.5 Hz, 3H), 4.03 (d, J = 3.6 Hz, 3H). ^13^C NMR (101 MHz, chloroform-d) δ 159.43, 156.84, 148.06, 144.61, 140.15, 136.27, 134.40, 131.03, 129.68, 125.42, 125.37, 123.64, 122.81, 117.75, 104.93, 77.23, 61.49, 56.49, 22.66, 14.13. HRMS (ESI) m/z calcd for C_17_H_13_ClNO_2_ [M + H]^+^: 298.0635, found 298.0629.

9-fluoro-5,6-dimethoxyindeno [1,2,3-ij]isoquinoline (**15**). Yield 44%; yellow solid; ^1^H NMR (400 MHz, chloroform-d) δ 8.61 (d, J = 5.8 Hz, 1H), 7.99 (t, J = 6.6 Hz, 1H), 7.86 (d, J = 8.5 Hz, 1H), 7.50 (d, J = 5.8 Hz, 1H), 7.16 (t, J = 8.9 Hz, 1H), 7.03 (s, 1H), 4.15 (s, 3H), 4.09 (s, 3H). ^13^C NMR (101 MHz, chloroform-d) δ 148.40, 136.31, 134.62, 130.11, 125.52, 123.72, 123.44, 117.97, 104.96, 77.25, 61.57, 56.61. HRMS (ESI) m/z calcd for C_17_H_13_FNO_2_ [M + H]^+^: 282.0929, found 282.0925.

8-fluoro-5,6-dimethoxyindeno [1,2,3-ij]isoquinoline (**16**). Yield 42%; yellow solid; ^1^H NMR (400 MHz, chloroform-d) δ 8.59 (d, J = 5.9 Hz, 1H), 8.23 (d, J = 6.9 Hz, 1H), 7.77 (d, J = 8.8 Hz, 1H), 7.53 (d, J = 5.8 Hz, 1H), 7.17 (t, J = 9.0 Hz, 1H), 7.11 (s, 1H), 4.20 (s, 3H), 4.13 (s, 3H). ^13^C NMR (101 MHz, chloroform-d) δ 159.42, 157.09, 147.63, 144.73, 133.91, 131.02, 125.69, 125.60, 125.54, 124.06, 117.76, 116.38, 116.15, 110.22, 109.98, 104.37, 77.23, 61.42, 56.45. HRMS (ESI) m/z calcd for C_17_H_13_FNO_2_ [M + H]^+^: 282.0929, found 282.0925.

5,6-dimethoxyindeno [1,2,3-ij]isoquinoline (**17**). Yield 38%; yellow solid; ^1^H NMR (400 MHz, chloroform-d) δ 8.61 (d, J = 5.8 Hz, 1H), 8.18 (d, J = 7.0 Hz, 1H), 8.07 (d, J = 7.1 Hz, 1H), 7.48 (q, J = 5.7, 4.1 Hz, 3H), 7.05 (s, 1H), 4.16 (s, 3H), 4.09 (s, 3H). 13C NMR (101 MHz, chloroform-d) δ 159.31, 158.36, 147.83, 144.73, 138.66, 138.08, 130.90, 130.01, 128.49, 126.51, 124.77, 123.35, 122.30, 117.20, 104.71, 77.22, 61.40, 56.43, 29.71. HRMS (ESI) m/z calcd for C_17_H_14_NO_2_ [M + H]^+^: 264.1023, found 264.1019.

### 3.2. Antibacterial Activities

The antibacterial activities were assessed using the minimum inhibitory concentration (MIC) method. Each compound was tested at concentrations of 256, 128, 64, 32, 16, 8, 4, 2, 1, and 0.5 μg/mL using the continuous dilution method in liquid media at specified temperatures. For the liquid media, beef extract peptone medium was prepared from beef extract (3.0 g/L), peptone (10 g/L), and NaCl (5 g/L) and adjusted to pH 7.2–7.4. The temperature was maintained at 37 °C. The positive control for Gram-positive bacteria (*S. aureus* and *B. subtilis*) was penicillin sodium, while for Gram-negative bacteria (*E. coli* and *P. aeruginosa*) it was streptomycin sulfate. The MIC was defined as the lowest concentration at which no microbial growth was observed.

### 3.3. Antitumor Activities

This experiment used PC-3 (prostate cancer cells) and MDA-MB-231 (triple-negative breast cancer cells), all of which were stored at the Institute of Basic Medical Sciences, Xi’an Medical University. The cells were cultured in DMEM medium and were maintained in a CO_2_ incubator at 5% CO_2_, 37 °C, and with saturated humidity. The medium was changed every 24 h, and passaging was performed. Finally, cells in the logarithmic growth phase were selected for the relevant experiments.

### 3.4. MTT Assay

The tumor cells in the logarithmic growth phase were adjusted to a cell concentration of 1 × 10^5^ cells per milliliter using DMEM medium. Subsequently, 100 μL of the cell suspension was seeded into each well of a 96-well plate. The seeded cells were then placed in a CO_2_ incubator with 5% CO_2_ and saturated humidity and incubated at 37 °C for 24 h. After incubation, the old culture medium was removed, and 100 μL of serum-free culture medium was added to each well. Then, 100 μL of the test compound and positive control compound solutions at different concentration gradients were added to the experimental group wells, with 3 parallel controls set for each group, including a blank well (medium (energy-chemical, Xi’an, China), MTT, DMSO) and a control well (untreated cells, medium (energy-chemical, Xi’an, China), MTT, DMSO). The cells were then further incubated for 48 h. Approximately 4 h before the end of the incubation, 20 μL of 5 mM MTT solution was added to each well, and the cells were incubated for an additional 4 h. After incubation, the supernatant was removed, and 100 μL of DMSO solution was added to each well. The 96-well plate was gently shaken on a shaker for 10 min, and the absorbance OD was measured at 570 nm using a microplate reader.

Cell inhibition rate (%) = (AC − AS/AC) × 100%

where AC is the absorbance of the control, and AS is the absorbance of the samples.

### 3.5. Molecular Modeling

The structure of FabH (PDB entry 1HNJ) was sourced from the RCSB Protein Data Bank (http://www.pdb.org/pdb/home/home.do (Accessed on 4 April 2021)). To investigate the interactions between the active compounds and the enzyme, AutoDock 4.2 was utilized. Prior to docking, all heteroatoms were removed from the 1HNJ.pdb file to ensure the receptor was devoid of any ligands. Additionally, water molecules were eliminated, and hydrogen atoms were added in standard geometry using AutoDock tools. The ligand file was processed using the Chem3D Ultra visualization program to achieve energy minimization and obtain a standard 3D structure in .pdb format. Docking simulations were executed with a search radius of 48 Å, centered at coordinates x = 26.819, y = 19.041, and z = 28.216, and a grid spacing of 0.375 Å. The Lamarckian genetic algorithm (LGA) was employed for the docking process. Visualization of the docking results was performed using PyMOL (version 0.99; DeLano Scientific, San Carlos, CA, USA) and LigPlot+ v1.4.5.

## 4. Conclusions

In summary, the novel = strategy was employed to synthesize the natural product sarumine (**12**) and a series of derivatives (**13**–**17**), and their antibacterial activity and inhibition mechanisms were investigated. The antibacterial activity tests revealed that the sarumine derivatives (**15**, **16**) exhibited moderate inhibitory activity. Notably, compound **12** demonstrated potent activity against *S. aureus* and *B*. *subtilis* with an MIC of 8 μg/mL, showing comparable inhibitory effects to the positive control. Molecular simulation studies indicated that sarumine (**12**) exhibited significant binding affinity to FabH. The inhibitory effect of the Cl was superior to the others on the structure, and the antitumor activity result also suggested that the inhibitory ability on PC-3 displayed by the derivatives of F and Cl substitutions on R_1_ was better than that of MDA-MB-231. These findings suggest that sarumine and its derivatives represent new and promising candidates for further study.

## Figures and Tables

**Table 1:** Investigation of the reaction conditions of the one-pot synthesis of sarumine.

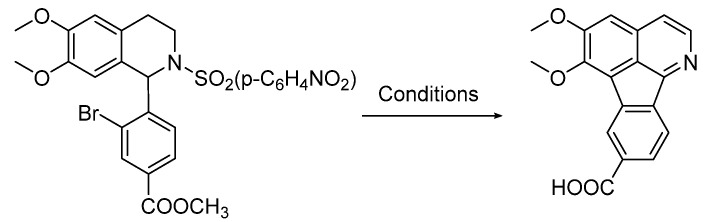					
Entry	Catalyst	Ligand	Solvent	T/C	Yield (%)
1	Pd(OAc)_2_	PPh_3_	DMSO	100	0
2	Pd(OAc)_2_	XPhos	DMSO	100	44
3	Pd(OAc)_2_	DavePhos	DMSO	100	55
4	Pd(OAc)_2_	DavePhos	DMSO	120	58
5	Pd(OAc)_2_	DavePhos	DMSO	135	71
6	Pd(OAc)_2_	DavePhos	DMSO	145	71
7	Pd(OAc)_2_	DavePhos	DMF	135	37
8	Pd(OAc)_2_	DavePhos	DCM	135	0
9	Pd(OAc)_2_	DavePhos	THF	135	12

**Table 2:** Antibacterial activities of compounds **12**–**17**.

Compd MIC (μg/mL)	*S. aureus*	*B. subtilis*	*P. aeruginosa*	*E. coli*
12 (Sarumine)	8	8	16	16
13	128	128	128	128
14	128	128	64	128
15	64	64	64	64
16	64	64	32	32
17	256	256	128	128
Penicillin sodium			16	16
Streptomycin sulfate	8	8		

**Table 3:** The binding free energy of sarumine derivatives with FabH.

Compound	Binding Free Energy (Kcal/mol)
12 (sarumine)	−9.885
13	−5.232
14	−6.097
15	−7.876
16	−7.994
17	−5.989

**Table 4:** Antitumor activity of sarumine derivatives according to PC-3 and MDA-MB-231 assays.

Sample	PC-3 IC_50_ μM	MDA-MB-231 IC_50_ μM
**12 (sarumine)**	20	32
**13**	15	10
**14**	7	9
**15**	49	66
**16**	60	206
**17**	204	236
Adriamycin	2.2	0.66

## Data Availability

The data are contained within this article or the Supplementary Materials.

## Supplementary Materials

The following supporting information can be downloaded at: https://www.mdpi.com/article/10.3390/ijms252212412/s1.
