# Supplementary material for: Antibacterial and Antitumor Activities of Synthesized Sarumine Derivatives
Source: Int J Mol Sci. 2024 Nov 19;25(22):12412. doi: 10.3390/ijms252212412 (PMC11595285; doi:10.3390/ijms252212412)
Supplement: Supplementary file 1 [file ijms-25-12412-s001.zip › ijms-3316690-supplementary.pdf]

Supporting information

Antibacterial and Antitumor Activities of Synthesized Sarumine Derivatives

Fangzhou Yang[1,2], Bin Jia, Hongli Wen, Xiufang Yang and Yangmin Ma *

1. [1] College of Chemistry and Chemical Engineering, Shaanxi University of Science & Technology. Key Laboratory of Chemical Additives for China National Light Industry, Xi’an 710021, China; fangzhouyang6@gmail.com (F.Y.); jiabin@sust.edu.cn (B.J.); whl1234567891106@163.com (H.W.); yangxf@sust.edu.cn (X.Y.)
2. [2] Department of Applied Biology and Chemical Technology, The Hong Kong Polytechnic University, Kowloon, Hong Kong SAR, P. R. China; The Hong Kong Polytechnic University Shenzhen Research Institute, Shenzhen 518057, P. R. China
3. ***** Correspondence: mayangmin@sust.edu.cn

4. Experimental Section

4.1. Synthesis

1.2.1. General Procedure for preparation of compounds 2-17

To a solution of 3,4-dihydroxybenzaldehyde (1.00 g, 7.24 mmol) in DMF (30mL), we added K_2CO_3 (10.3 g, 72.4 mmol) and MeI (5.00 g, 36.2 mmol), which we then stirred at 90 °C for 1 h in an oil bath. The solution was filtrated and evaporated in vacuo. The residue was mixed with EtOAc and water; the organic layer was washed with water and 1M NaOH, then evaporated in vacuo. The residue was purified by column chromatography on silica gel (hexane/EtOAc, 4:1) to produce compound 2 (1.13 g, 94%) as a white solid.

Compound **2** (1 g, 6 mmol), NH_4OAc (240 mg, 3.1 mmol), and acetic acid (20 mL) were loaded in a 100 mL two-neck flask. Then, CH_3NO_2 (1 g, 16.3 mmol) was added

dropwise, and the mixture was stirred at 100 °C for 12 h in an oil bath. After cooling to the room temperature, 30 mL of water was added to the mixture. The resulting solid was filtered, washed with 6 mL of water, and vacuum-dried at 60 °C. Compound **3** was obtained with 93% yield as a yellow solid.

A 250 mL flask containing a stirring bar was dried with a heat-gun then cooledto the room temperature. Compound **3** (100 mg, 0.48 mmol) was dissolved in te THF (7 mL), and $LiAlH_4$ (63.6 mg, 1.65 mmol) was added into the solution slowly at 0 °C. This reactive mixture was stirred at 0 °C for 1 h, then heated to 70 °C for 0.5 h. Once there was no gas released, the mixture was cooled to 0 °C and then 1.5 mL water and 15 wt. % aqueous NaOH (1 mL) were added and extracted with CH_2Cl_2 (20 mL×3). The combined organic layers were washed with water, dried with $MgSO_4$, filtered, and concentrated under reduced pressure to give a colorless oil with 89% yield.

Compound **4** (1.47 g, 8.1 mmol) was dissolved in CH_2Cl_2 (15 mL), and Et_3N (1.2 g, 5.4 mmol) was added dropwise. Then, the mixture was added to p-nitrobenzenesulfonylchloride (0.55 g, 8.1 mmol) in CH_2Cl_2 (15 mL). After stirring at room temperature for 10 h, the reactive mixture was added to water (20 mL) and extracted by CH_2Cl_2 (30 mL×3), The combined organic layers were washed with water, dried with $MgSO_4$, filtered, and concentrated under reduced pressure. Then, the white solid was recrystallized by adding EtOH and n-hexane with 95% yield.

1.2.1.1. 3,4-Dimethoxybenzaldehyde (2)

Yield 94%; white solid; ^{1}H NMR (400 MHz, chloroform-*d*): 3.94 (s, 3H), 3.97 (s, 3H), 6.97 (d, 1H), 7.41 (s, 1H), 7.46 (d, 1H), 3.21 (s, 1H). ^{13}C NMR (101 MHz, chloroform-*d*) δ 190.76, 154.51, 149.66, 130.18, 126.71, 110.45, 109.07, 56.13, 55.97.

1.2.1.2. (E)-1,2-dimethoxy-4-(2-nitrovinyl)benzene (3)

Yield 93%; yellow solid; ^{1}H NMR (400 MHz, chloroform-*d*) δ 8.24 (d, *J* = 13.7 Hz, 1H), 7.79 (d, *J* = 13.8 Hz, 1H), 7.19 – 7.05 (m, 4H), 3.96 (d, *J* = 11.0 Hz, 6H). ^{13}C NMR (101 MHz, chloroform-*d*) δ 153.21, 149.52, 138.56, 134.65, 124.46, 124.18, 121.44,

115.96, 61.14, 55.99.

1.2.1.3. 2-(3,4-dimethoxyphenyl)ethan-1-amine (4)

Yield 89%; colorless oil; ^{1}H NMR (400 MHz, chloroform-*d*) δ 6.85 – 6.78 (m, 1H), 6.75 (d, *J* = 1.9 Hz, 1H), 6.73 (s, 1H), 3.86 (d, *J* = 7.2 Hz, 6H), 2.94 (t, *J* = 6.8 Hz, 2H), 2.69 (t, *J* = 6.8 Hz, 2H). ^{13}C NMR (101 MHz, chloroform-*d*) δ 148.90, 147.41, 132.45, 120.64, 112.14, 111.42, 55.80, 55.70, 43.56, 39.53.

1.2.1.4. N-(3,4-dimethoxyphenethyl)-4-nitrobenzenesulfonamide (5)

Yield 95%; white solid; ^{1}H NMR (400 MHz, chloroform-d) δ 8.36 – 8.30 (m, 2H), 8.01 – 7.94 (m, 2H), 6.78 (d, J = 8.0 Hz, 1H), 6.67 – 6.60 (m, 2H), 4.77 (t, J = 6.1 Hz, 1H), 3.86 (d, J = 10.0 Hz, 6H), 3.32 (q, J = 6.5 Hz, 2H), 2.78 (t, J = 6.7 Hz, 2H). ^{13}C NMR (101 MHz, $CDCl_3$) δ 150.02, 149.28, 148.23, 145.95, 129.63, 128.18, 124.27, 120.76, 111.98, 111.60, 77.35, 77.03, 76.71, 55.95, 55.91, 44.50, 35.49.

4.2. NMR Spectra of Compounds

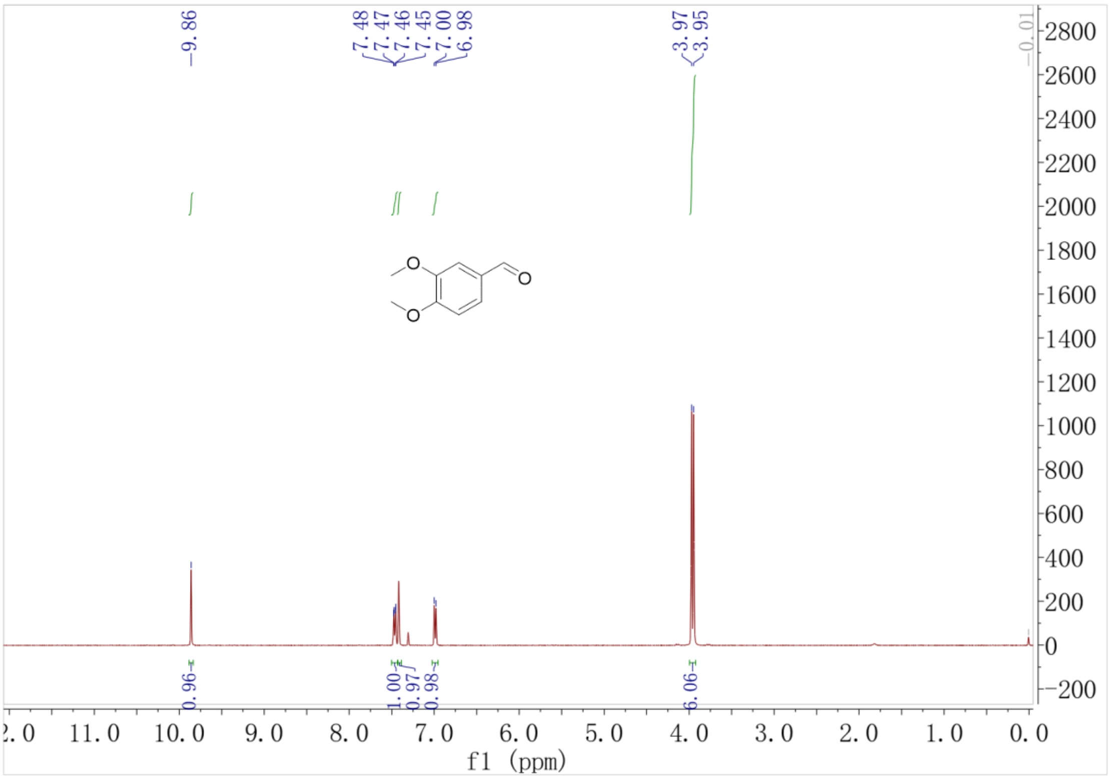

Figure S1. H and 13C NMR spectra of 2–17.

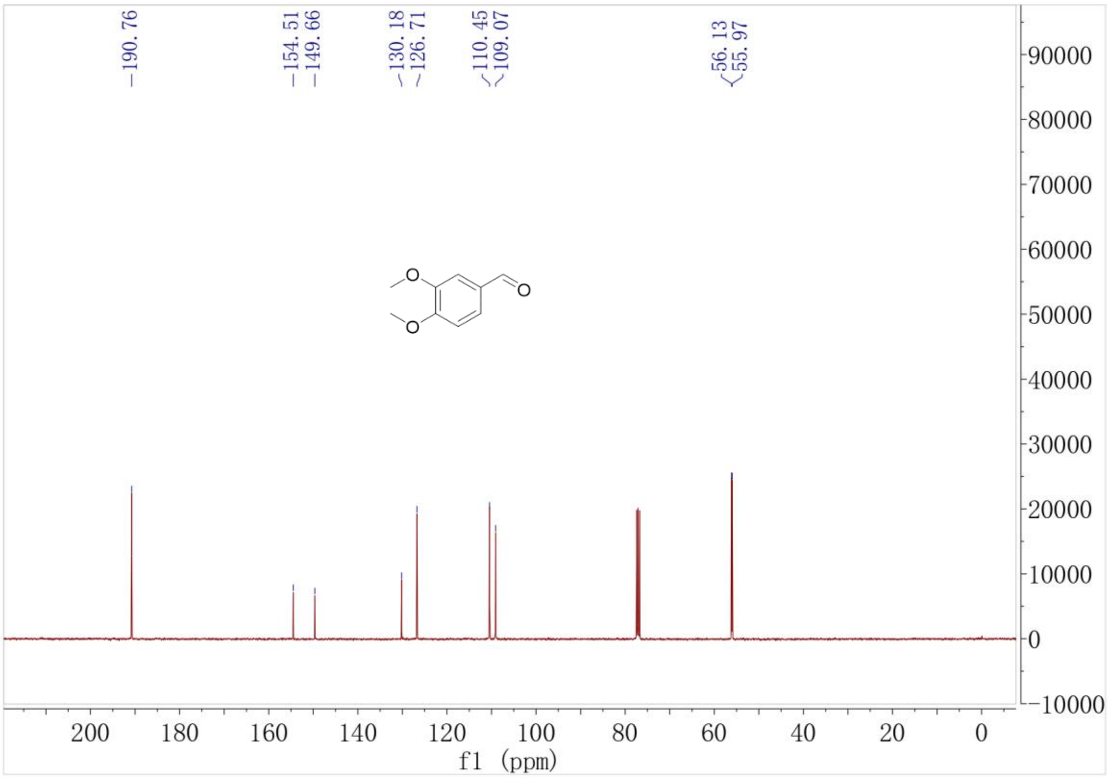

Figure S1. H and 13C NMR Spectra of 2–17.

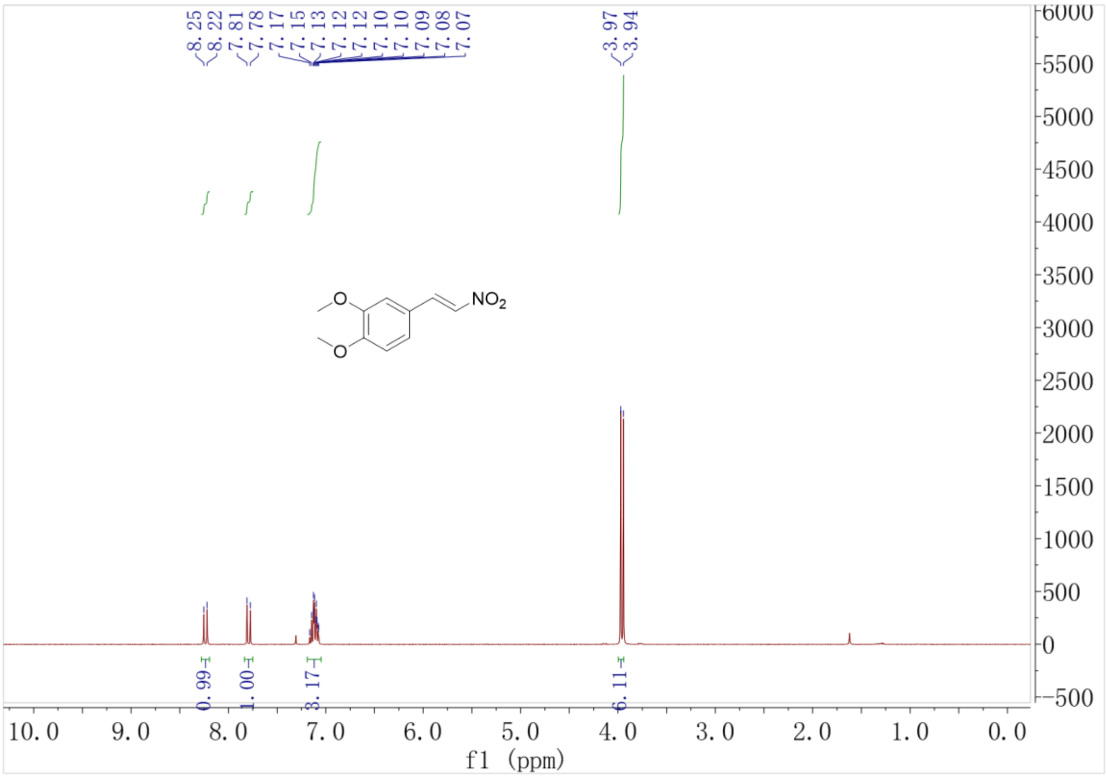

Figure S2. H and 13C NMR spectra of 2–17.

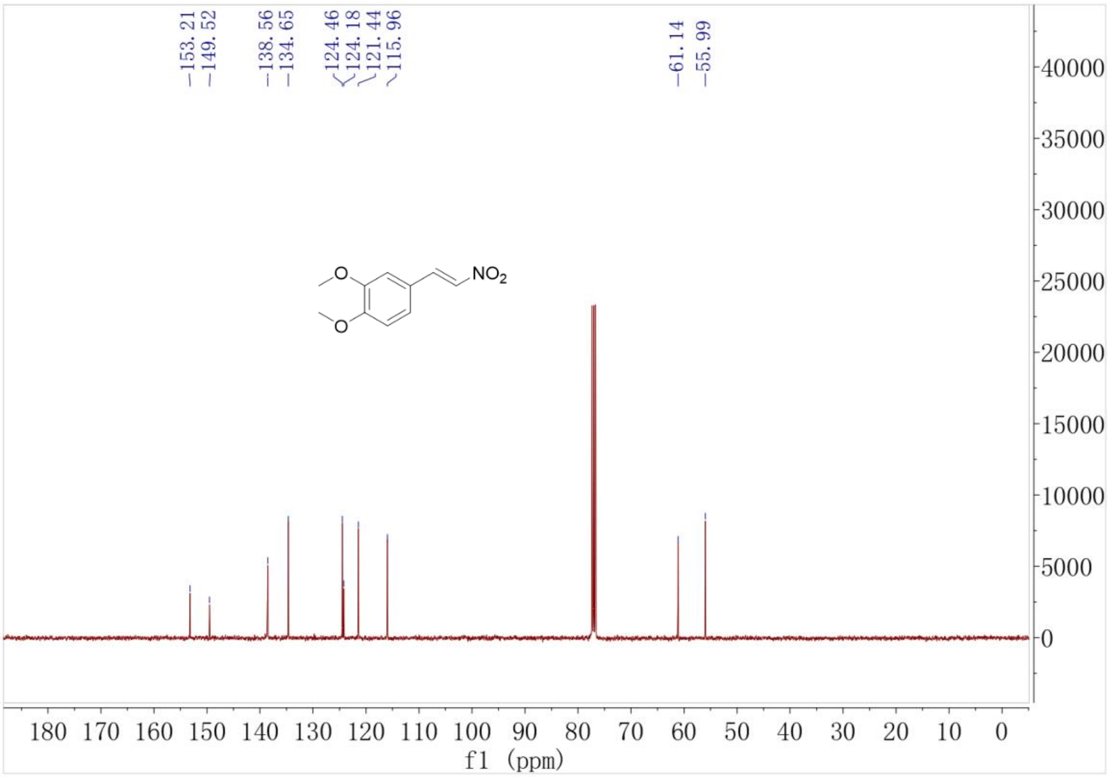

Figure S2. H and 13C NMR spectra of 2–17.

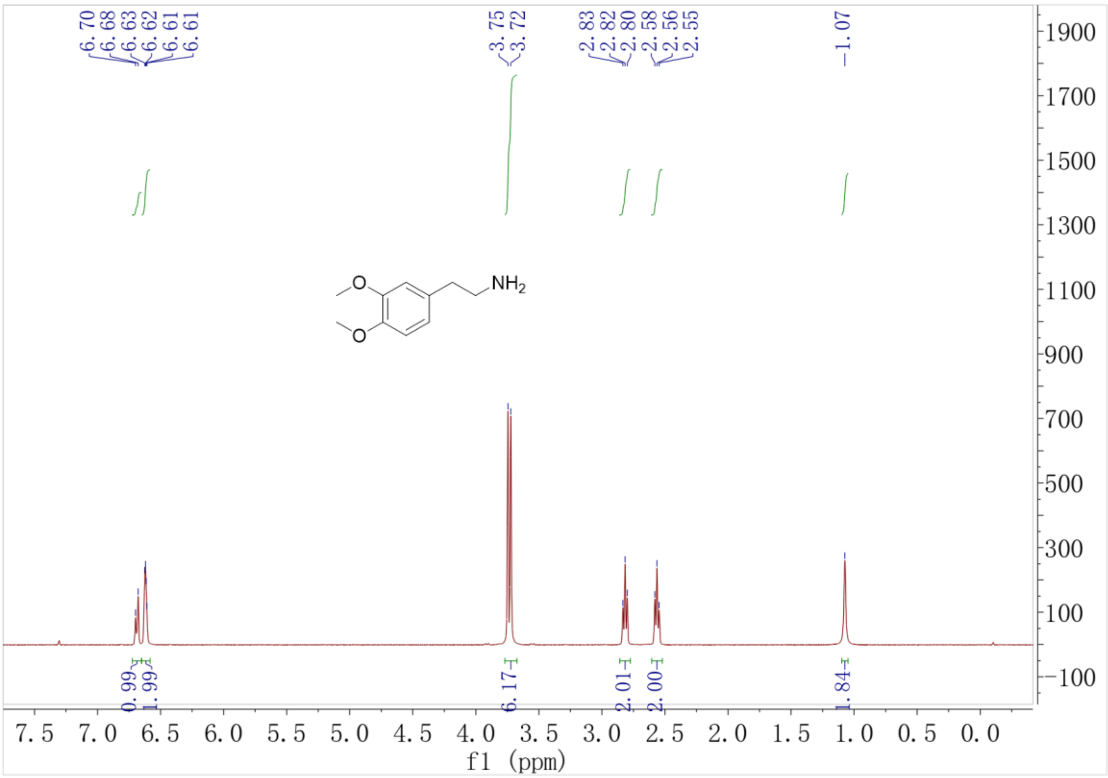

Figure S3. H and 13C NMR spectra of 2–17.

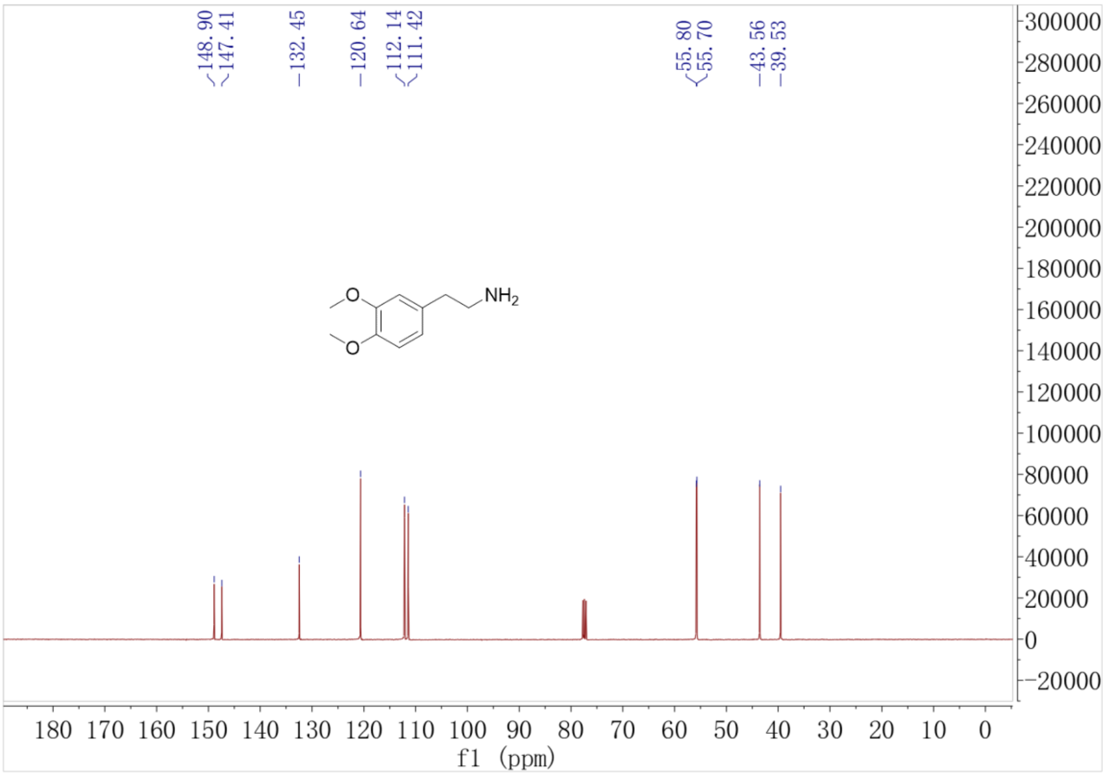

Figure S3. H and 13C NMR spectra of 2–17.

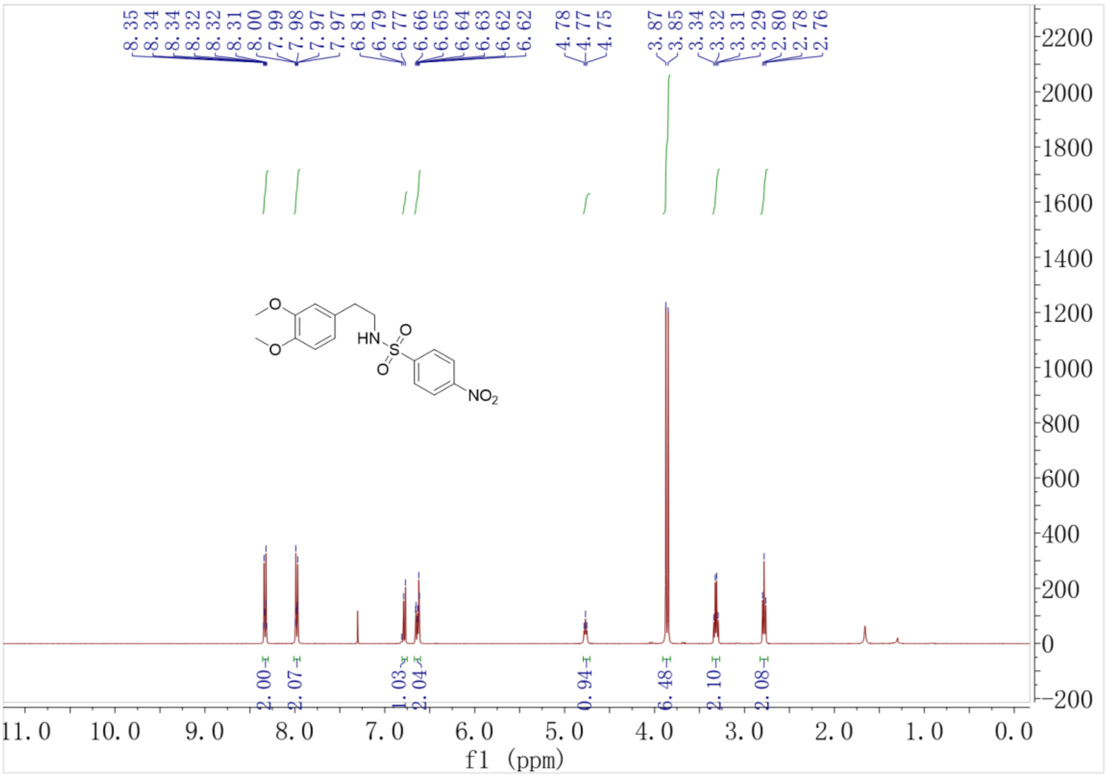

Figure S4. H and 13C NMR spectra of 2–17.

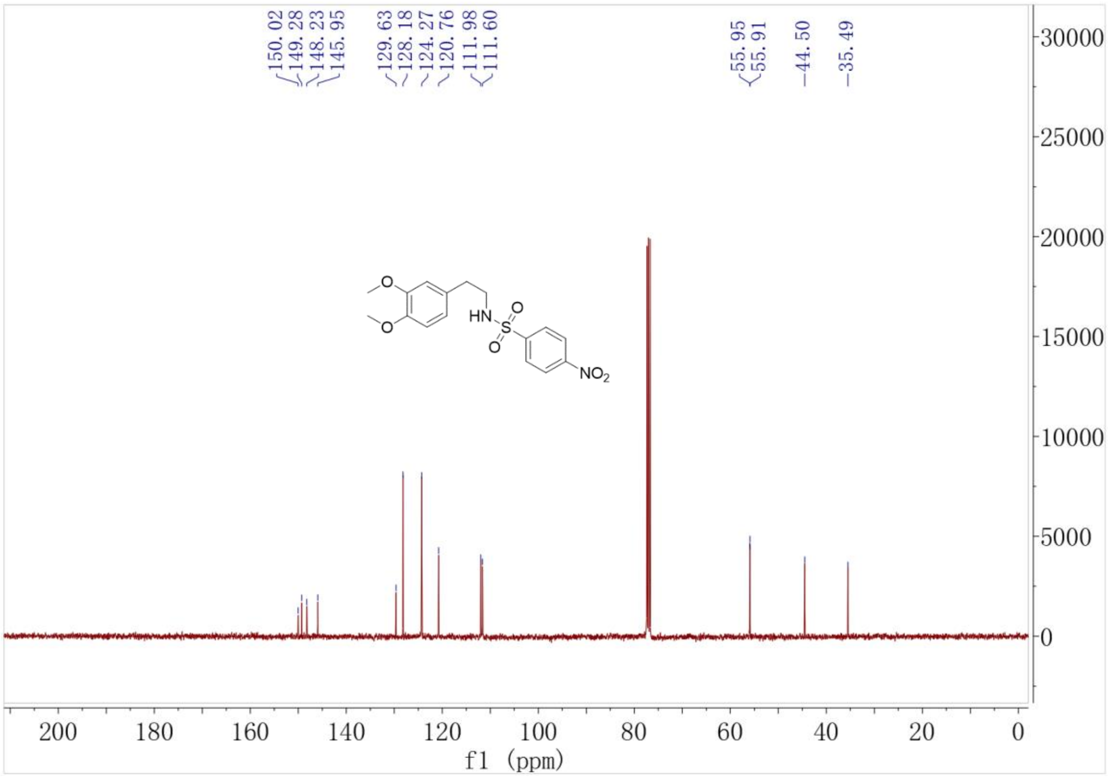

Figure S4. H and 13C NMR spectra of 2–17.

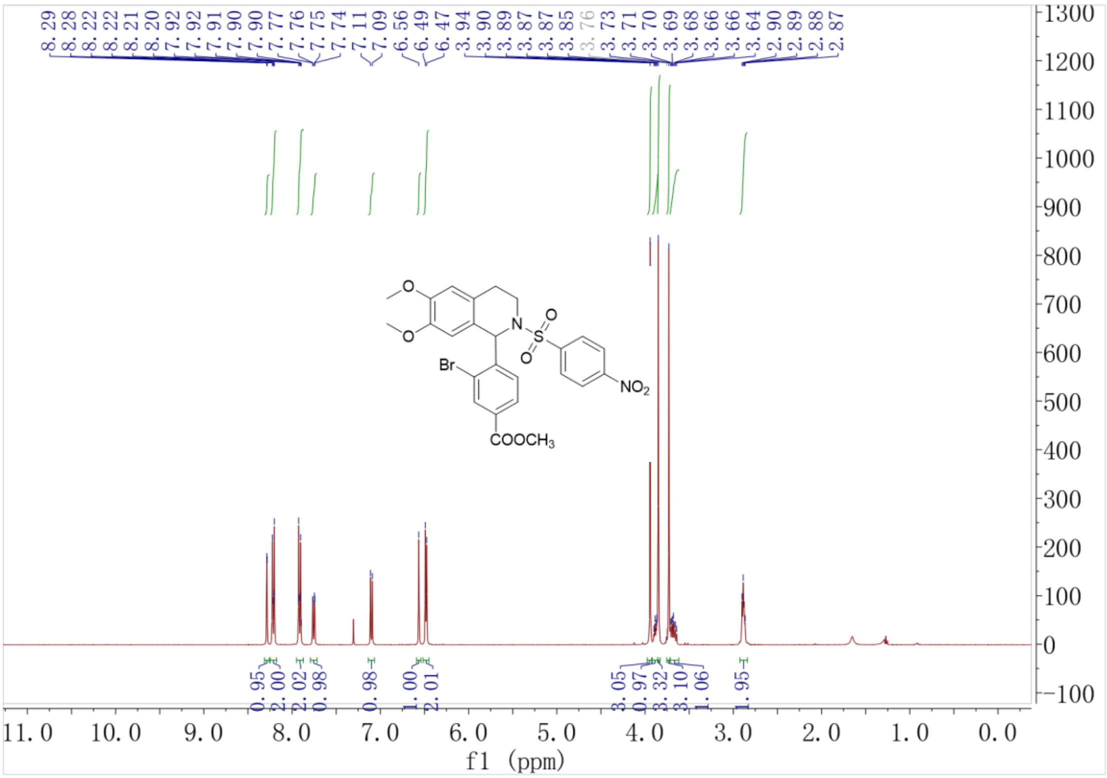

Figure S5. H and 13C NMR spectra of 2–17.

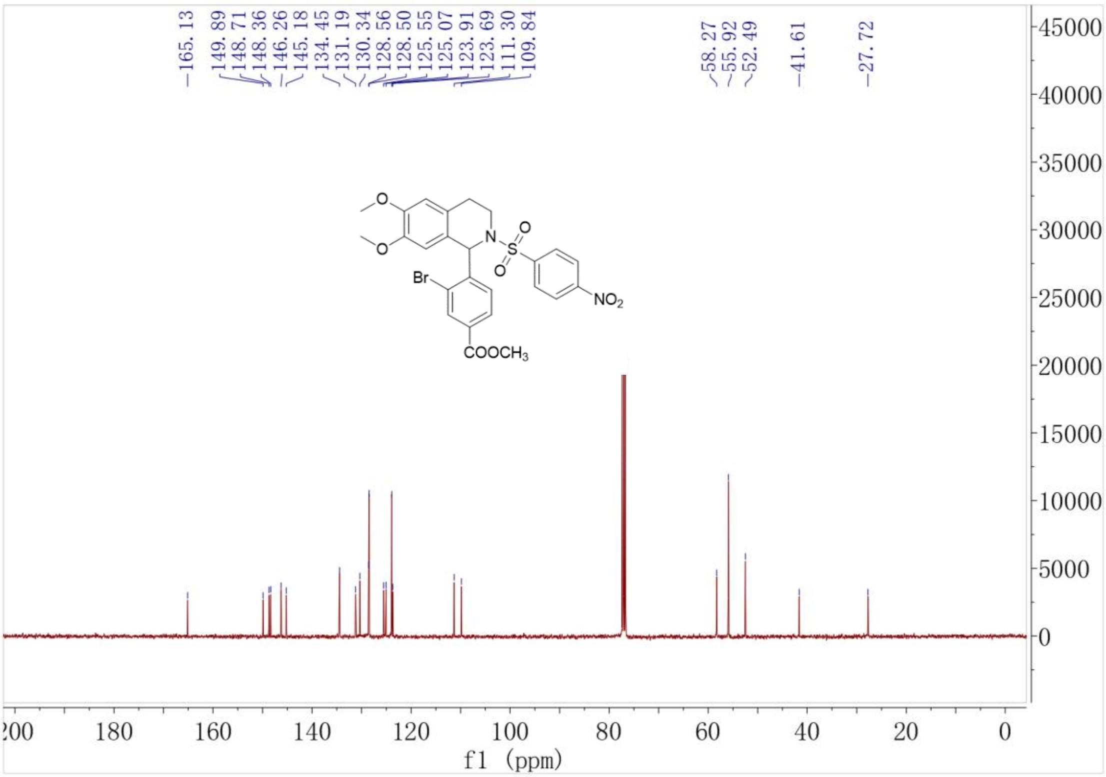

Figure S5. H and 13C NMR spectra of 2–17.

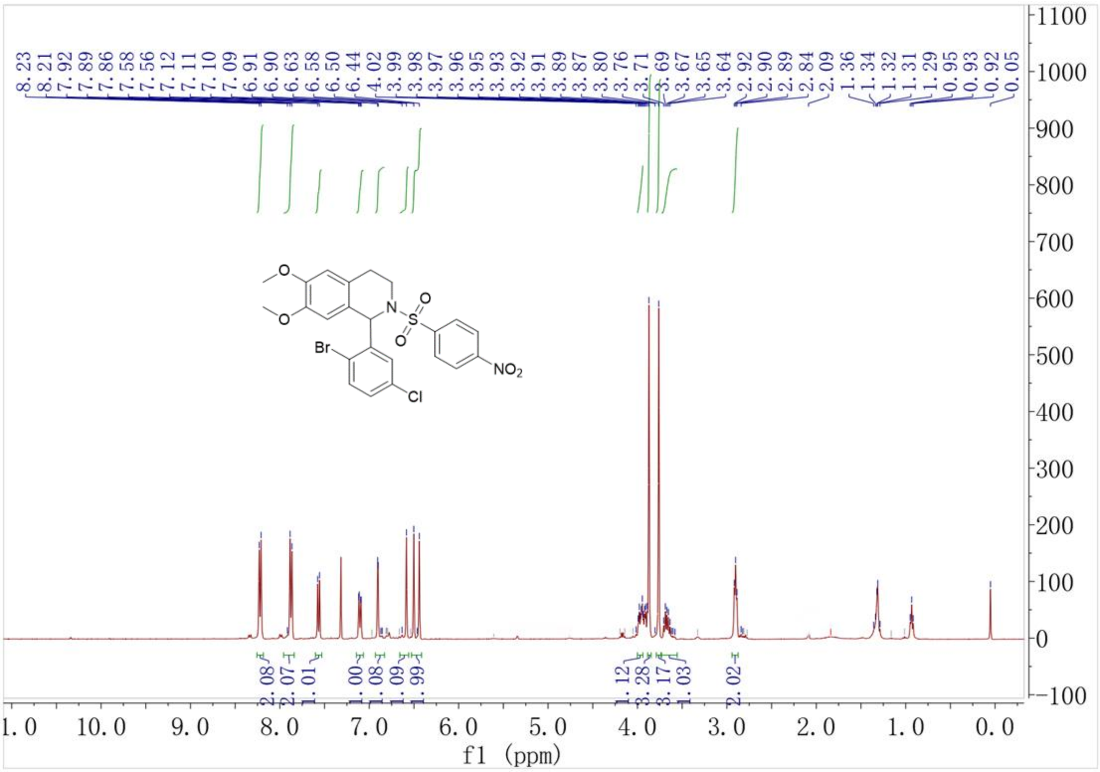

Figure S6. H and 13C NMR spectra of 2–17.

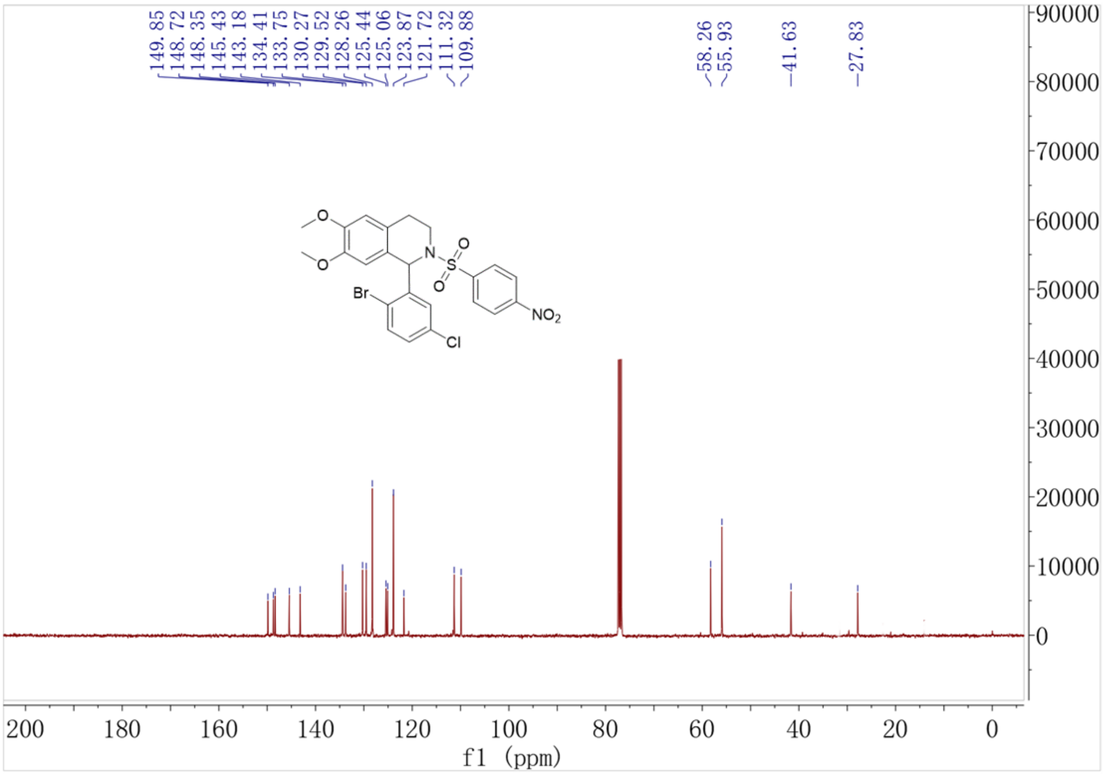

Figure S6. H and 13C NMR spectra of 2–17.

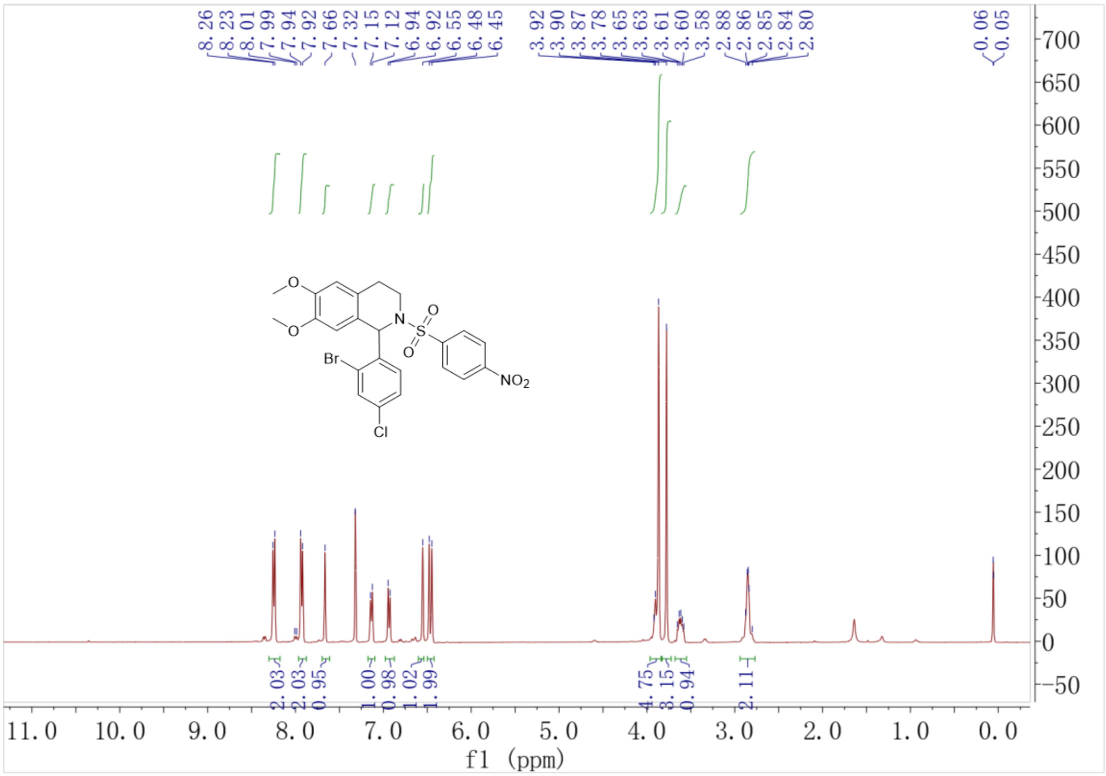

Figure S7. H and 13C NMR spectra of 2–17.

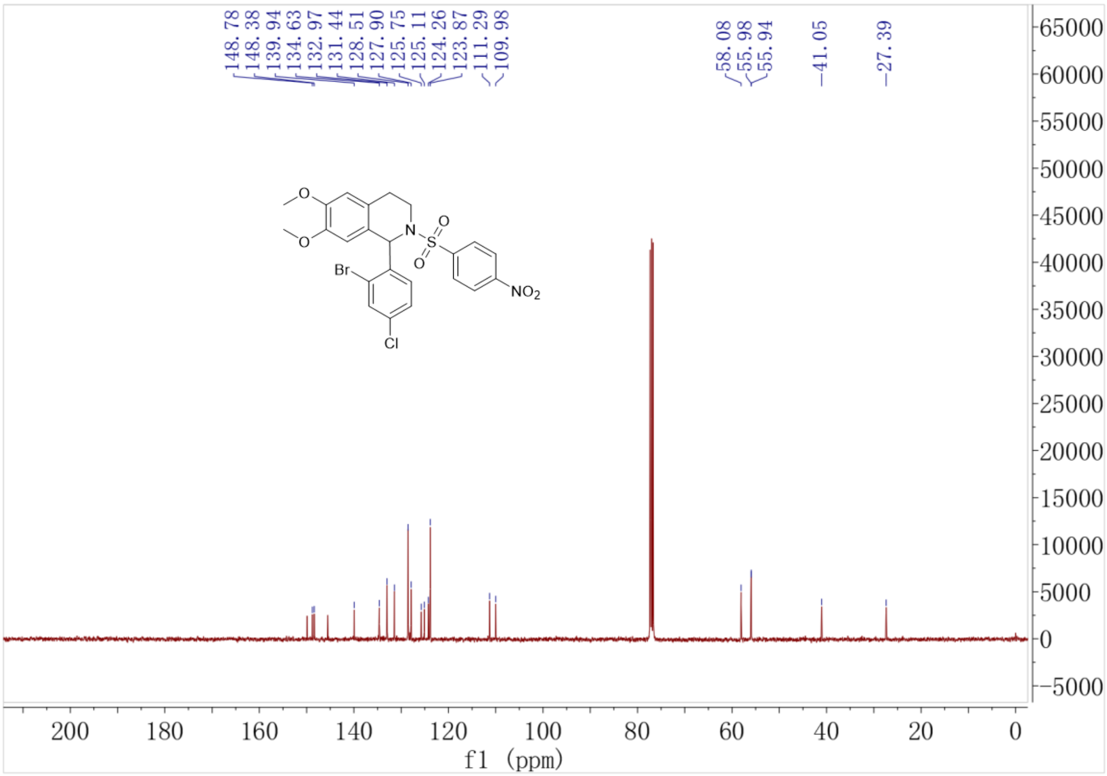

Figure S7. H and 13C NMR spectra of 2–17.

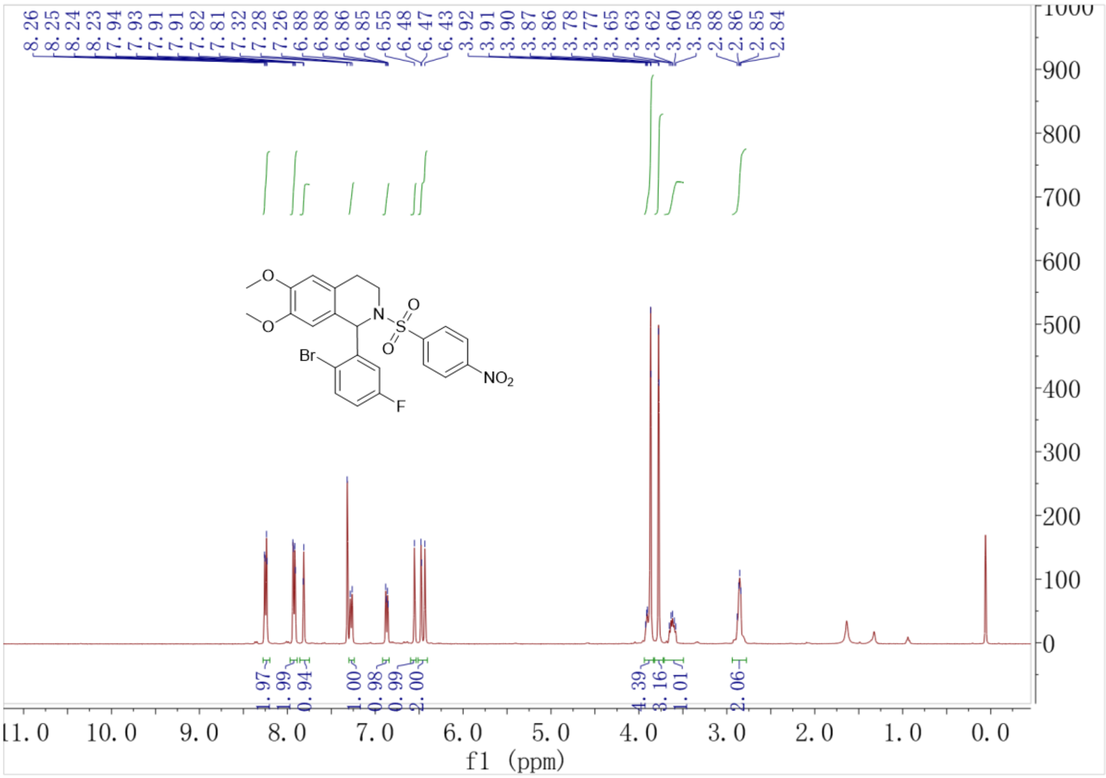

Figure S8. H and 13C NMR spectra of 2–17.

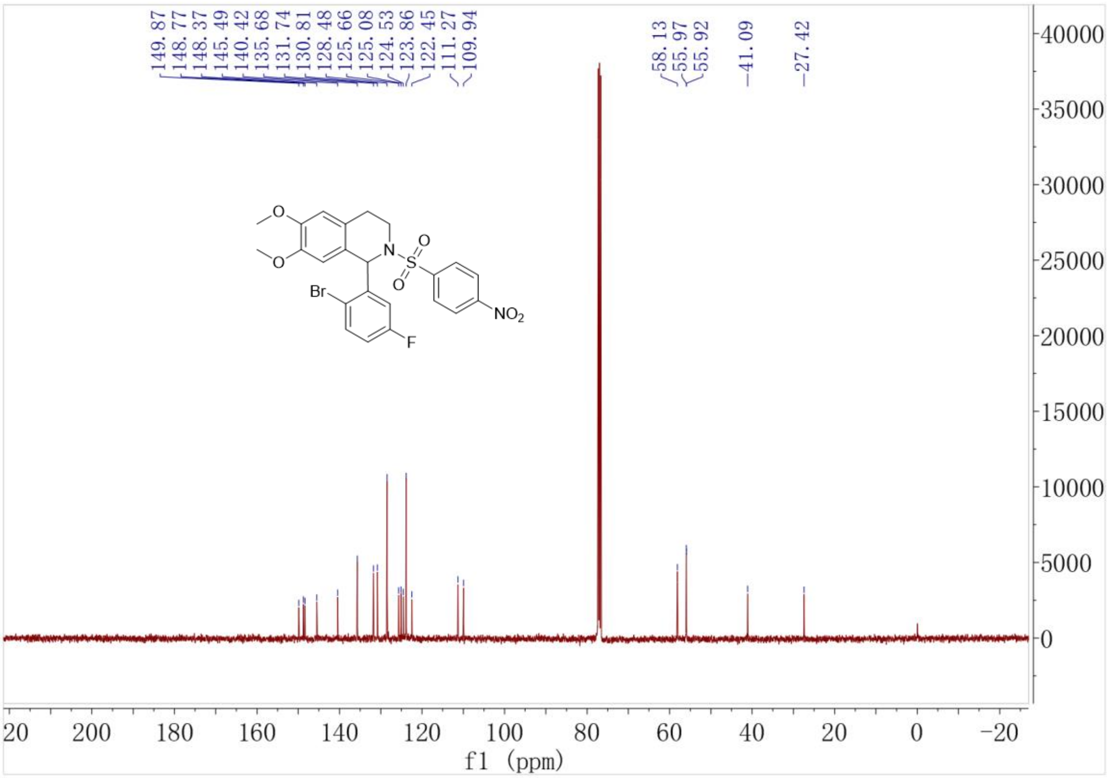

Figure S8. H and 13C NMR spectra of 2–17.

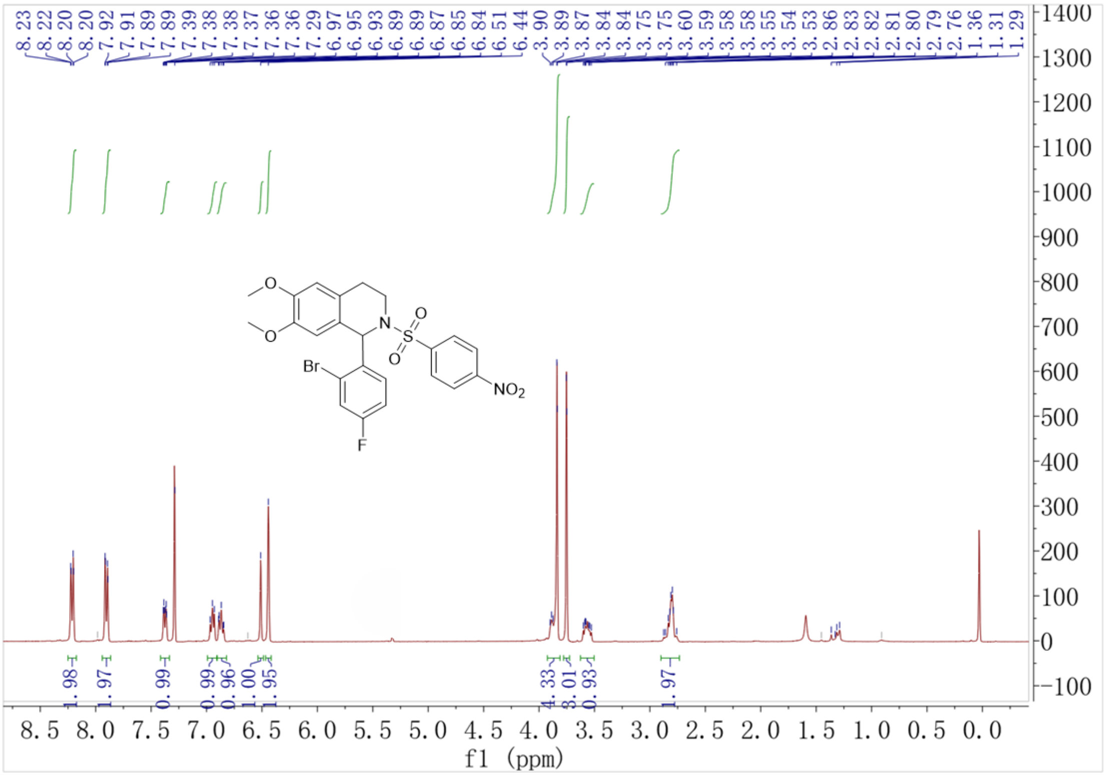

Figure S9. H and 13C NMR spectra of 2–17.

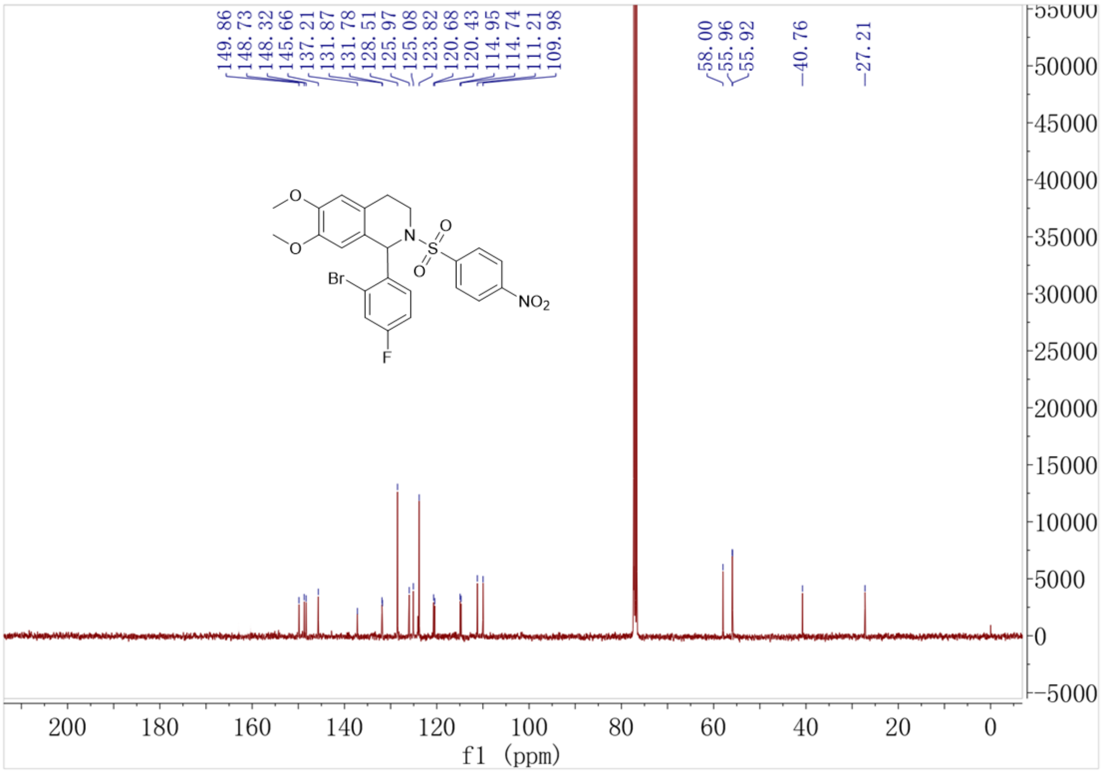

Figure S9. H and 13C NMR spectra of 2–17.

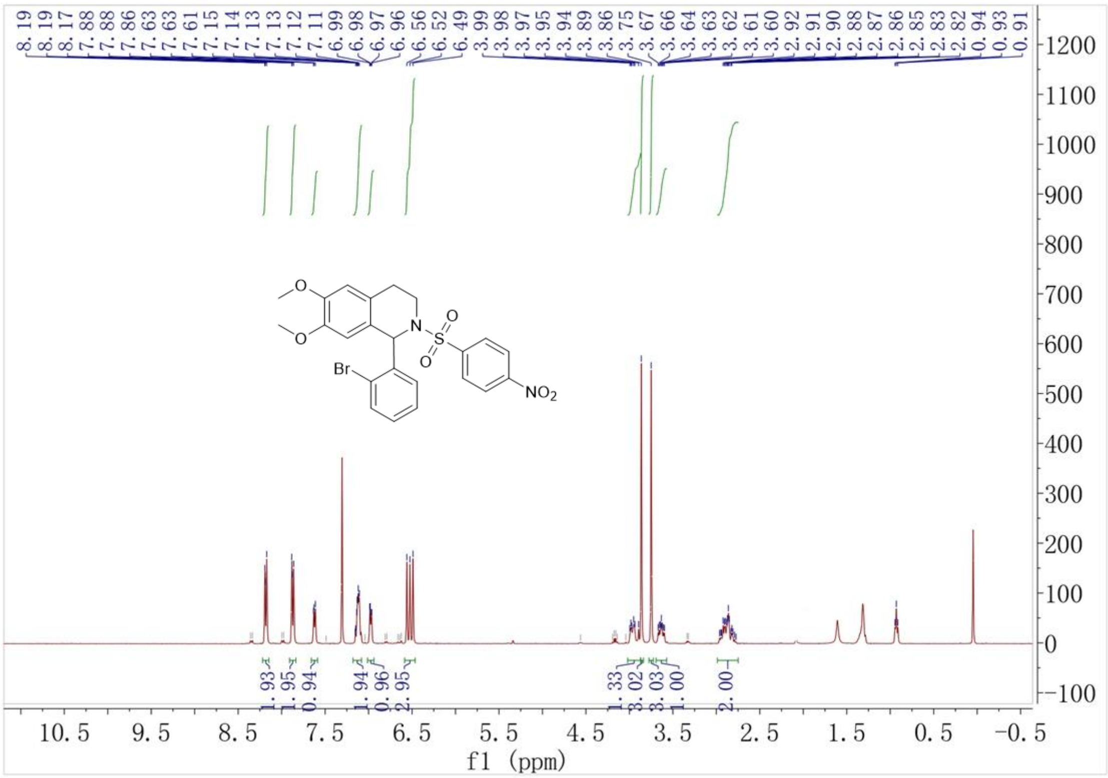

Figure S10. H and 13C NMR spectra of 2–17.

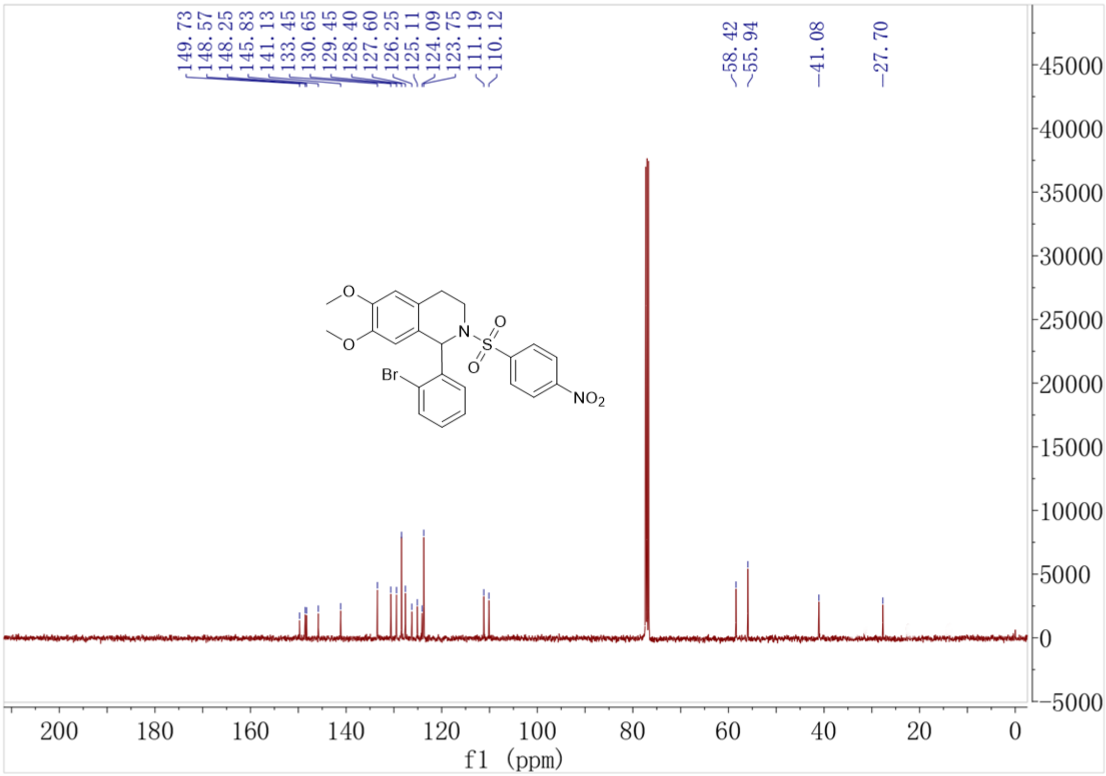

Figure S10. H and 13C NMR spectra of 2–17.

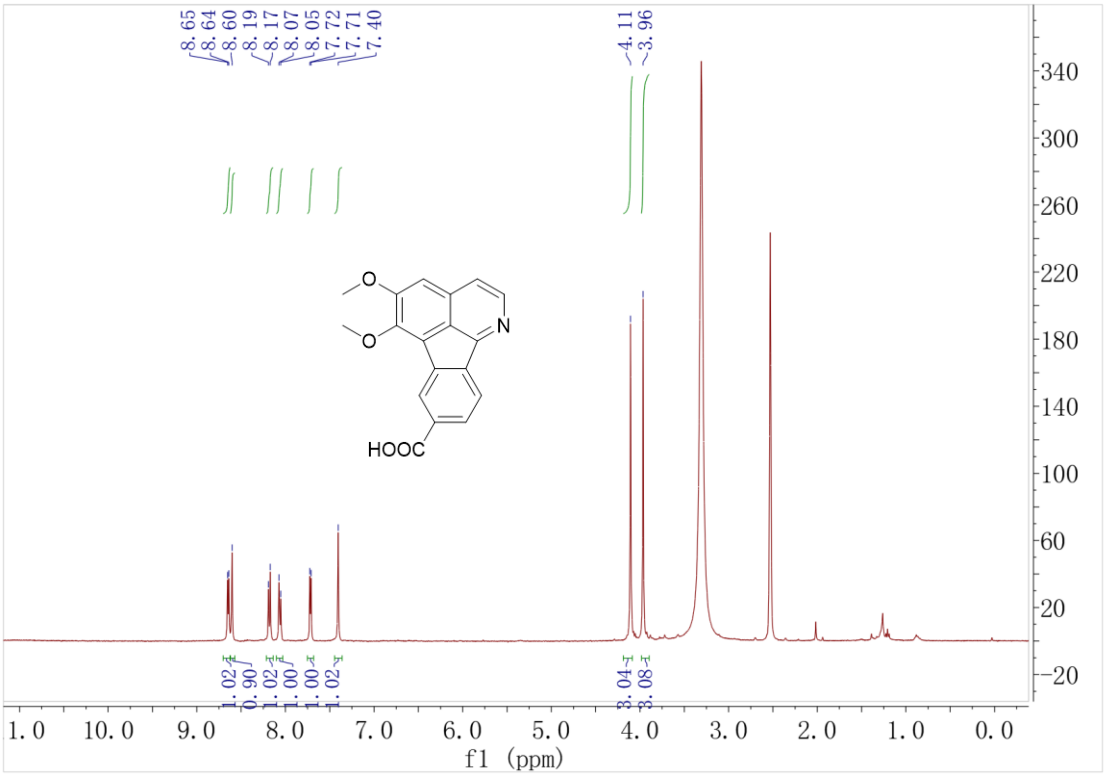

Figure S11. H and 13C NMR spectra of 2–17.

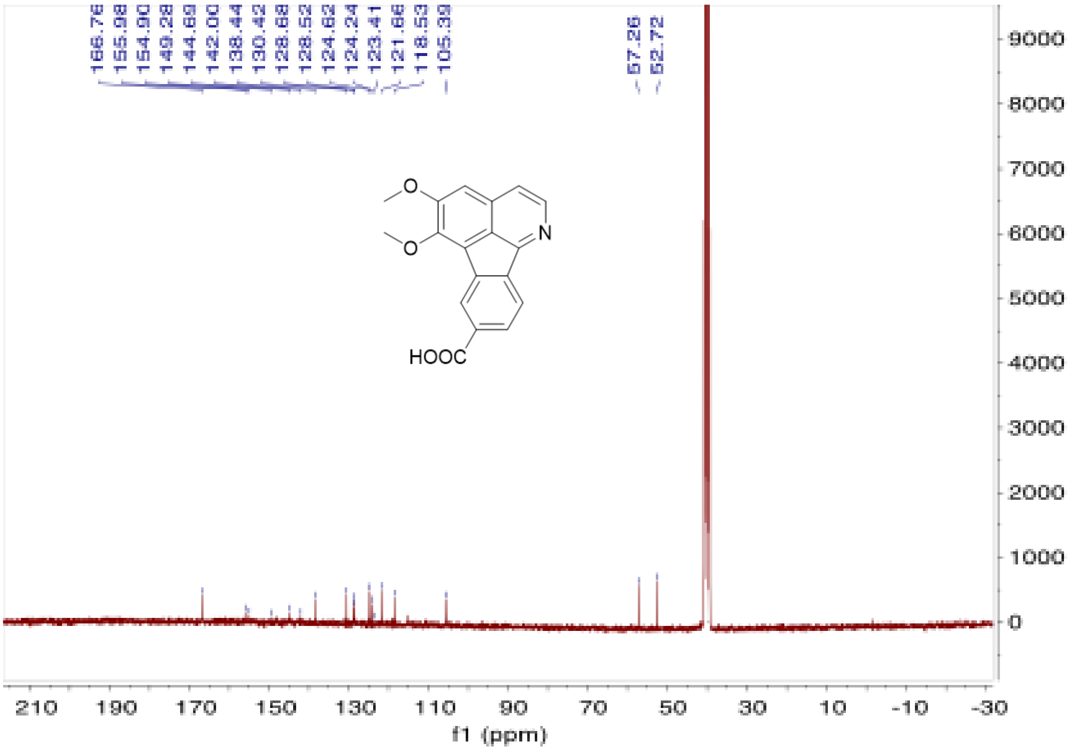

Figure S11. H and 13C NMR spectra of 2–17.

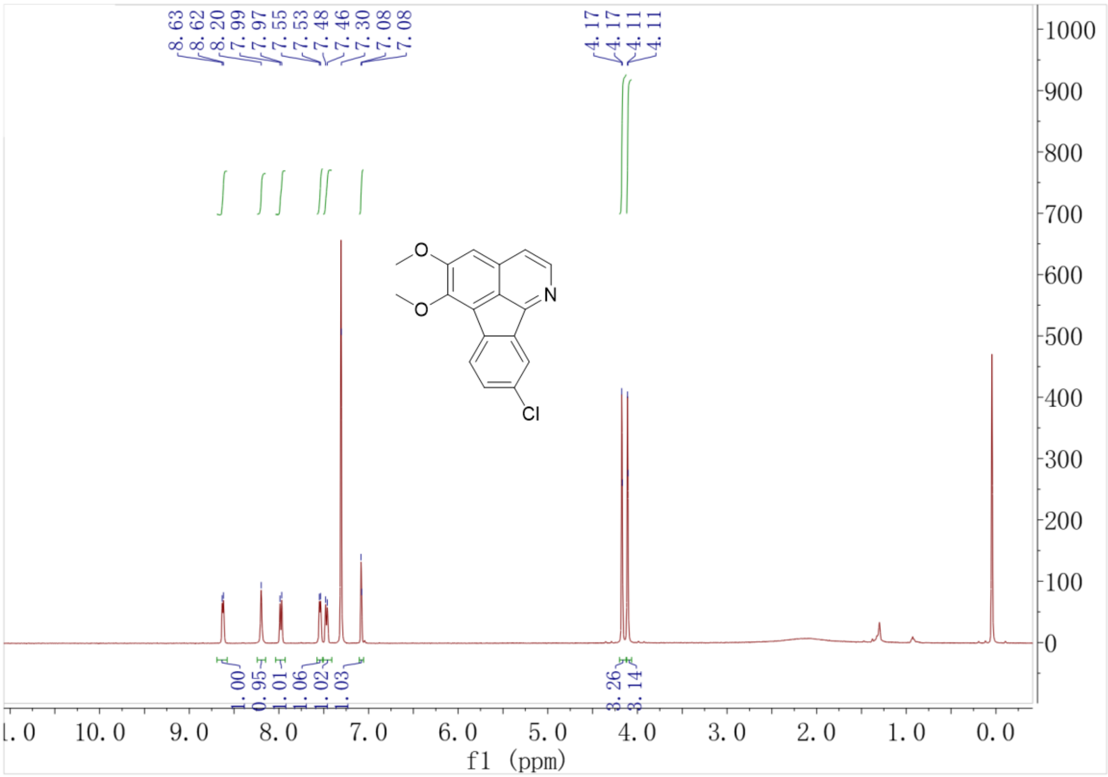

Figure S12. H and 13C NMR spectra of 2–17.

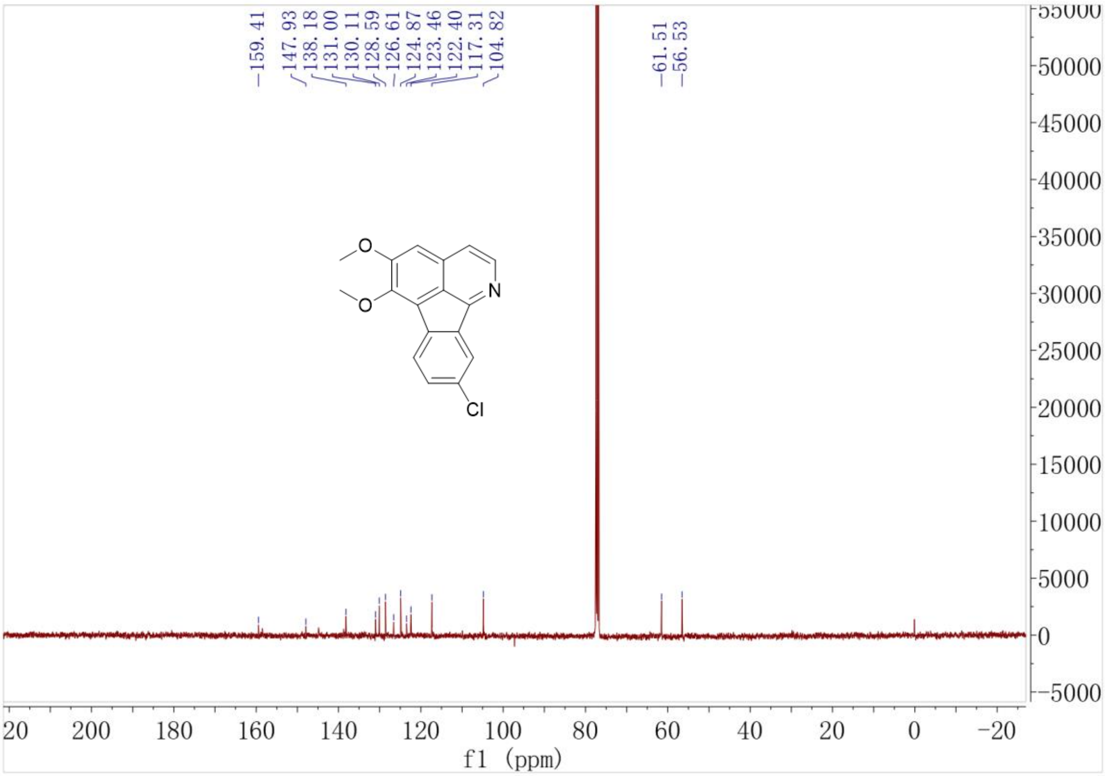

Figure S12. H and 13C NMR spectra of 2–17.

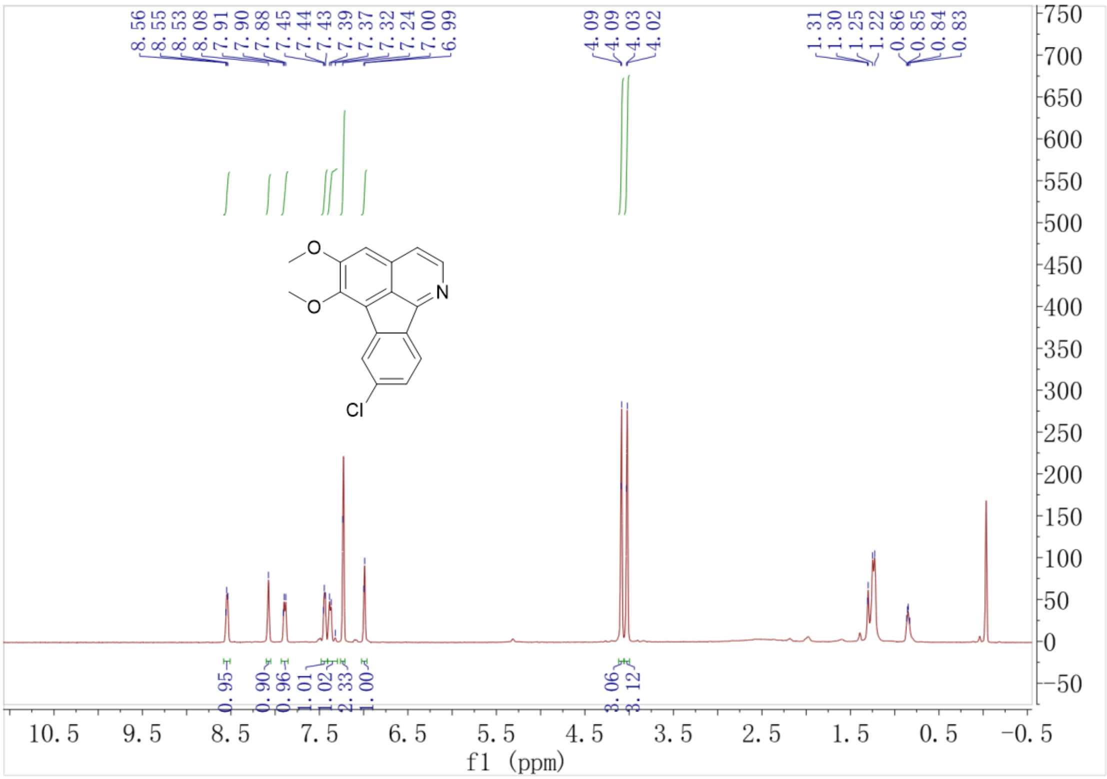

Figure S13. H and 13C NMR spectra of 2–17.

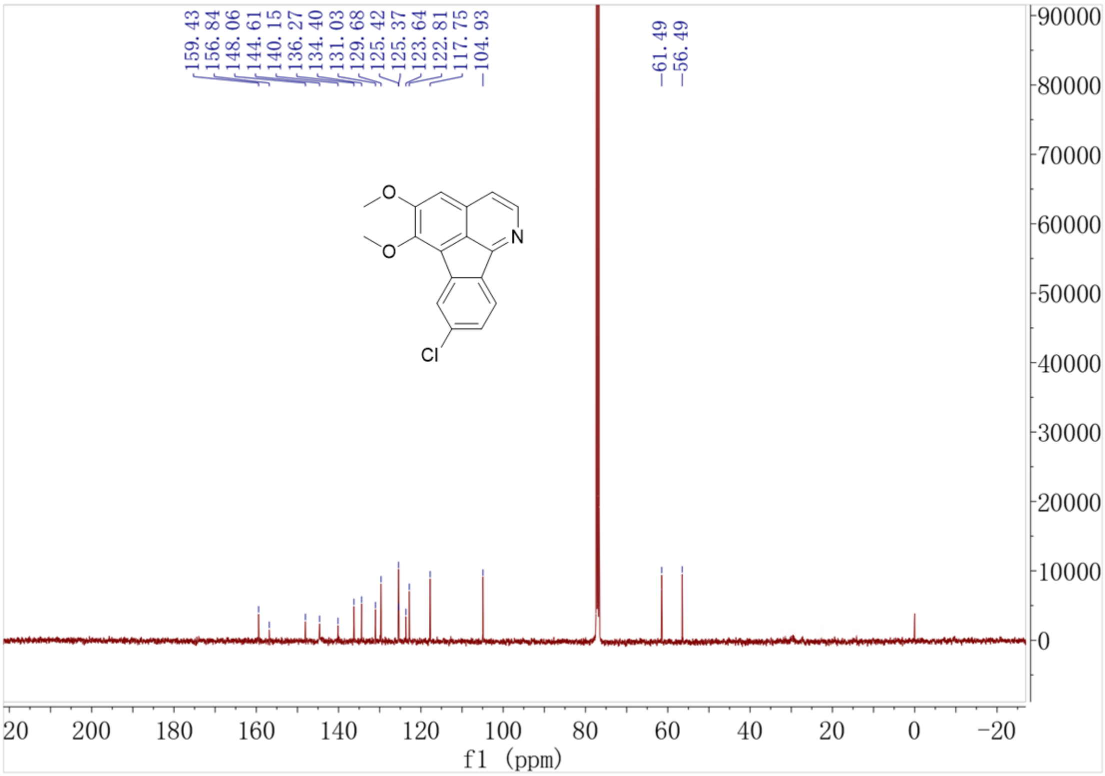

Figure S13. H and 13C NMR spectra of 2–17.

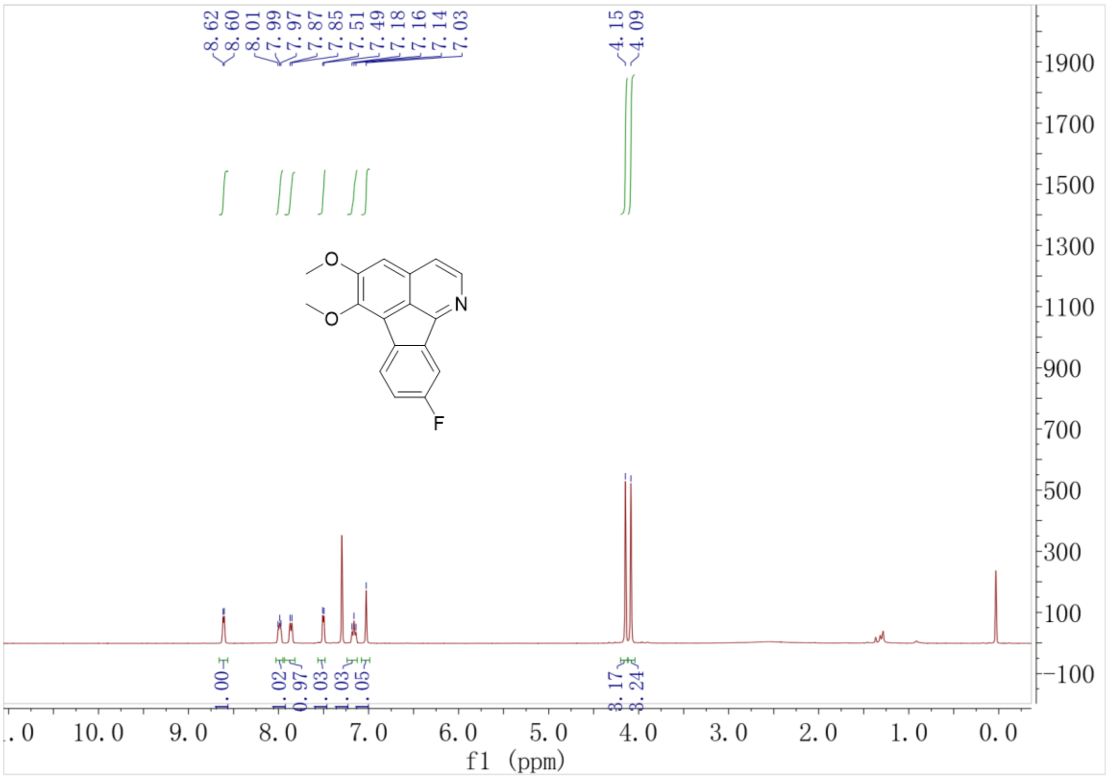

Figure S14. H and 13C NMR spectra of 2–17.

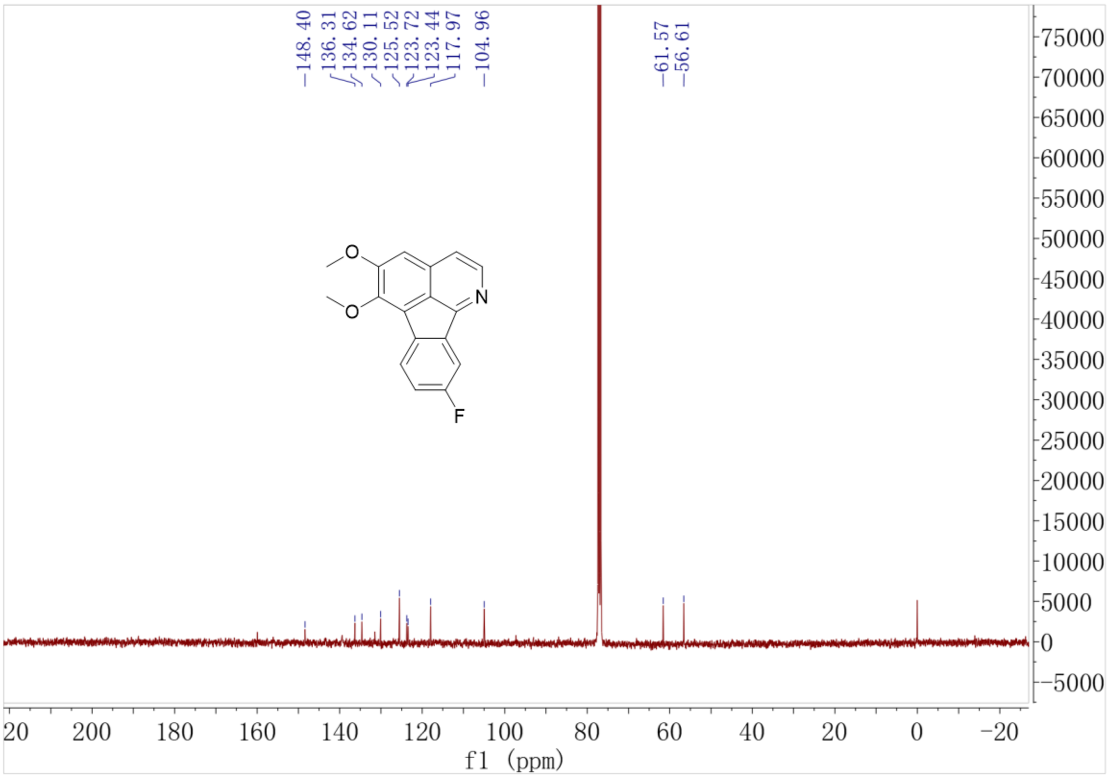

Figure S14. H and 13C NMR spectra of 2–17.

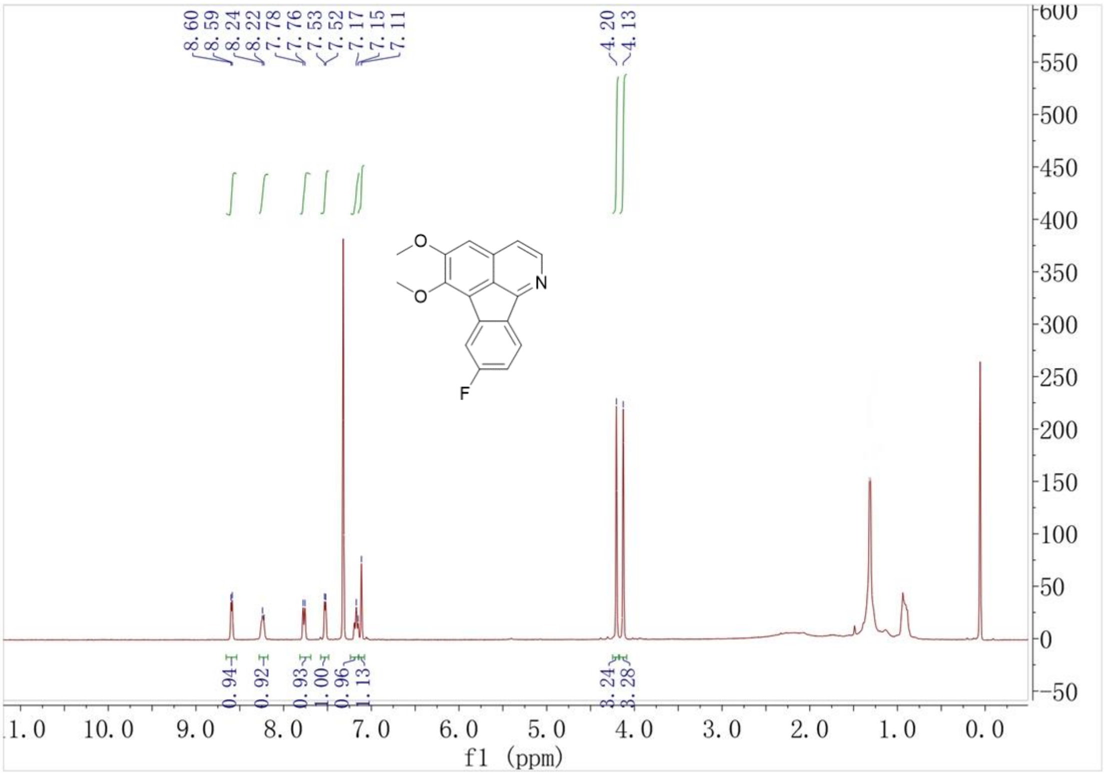

Figure S15. H and 13C NMR spectra of 2–17.

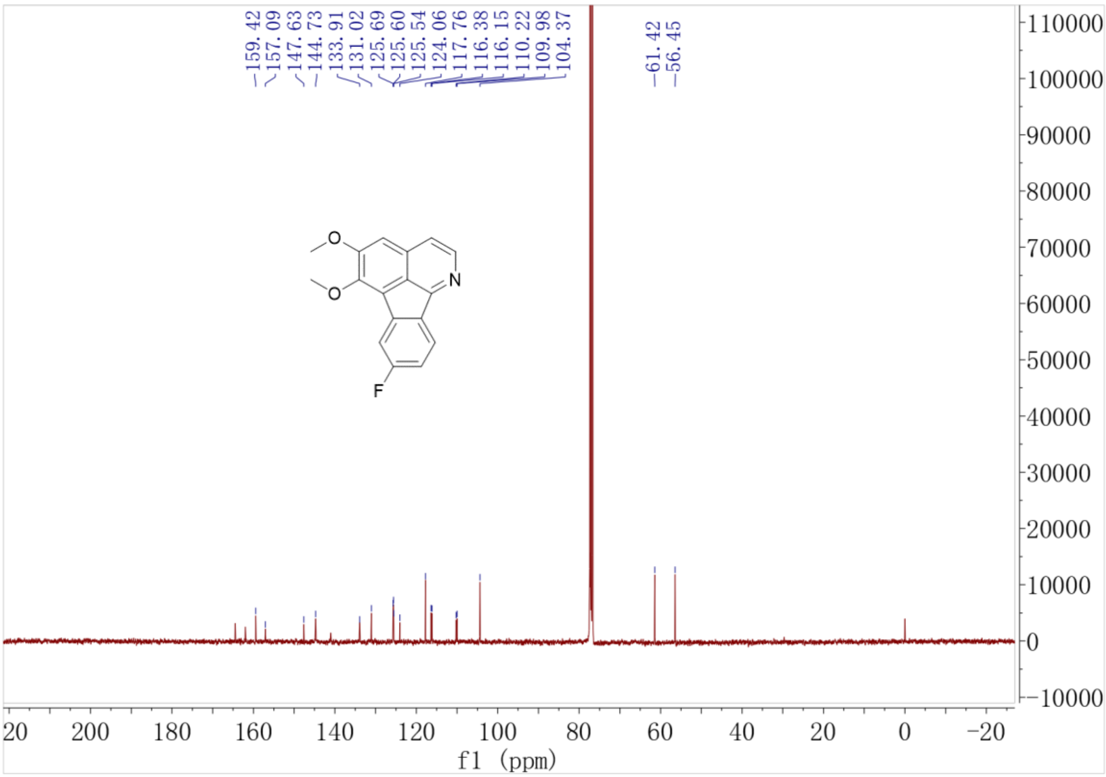

Figure S15. H and 13C NMR spectra of 2–17.

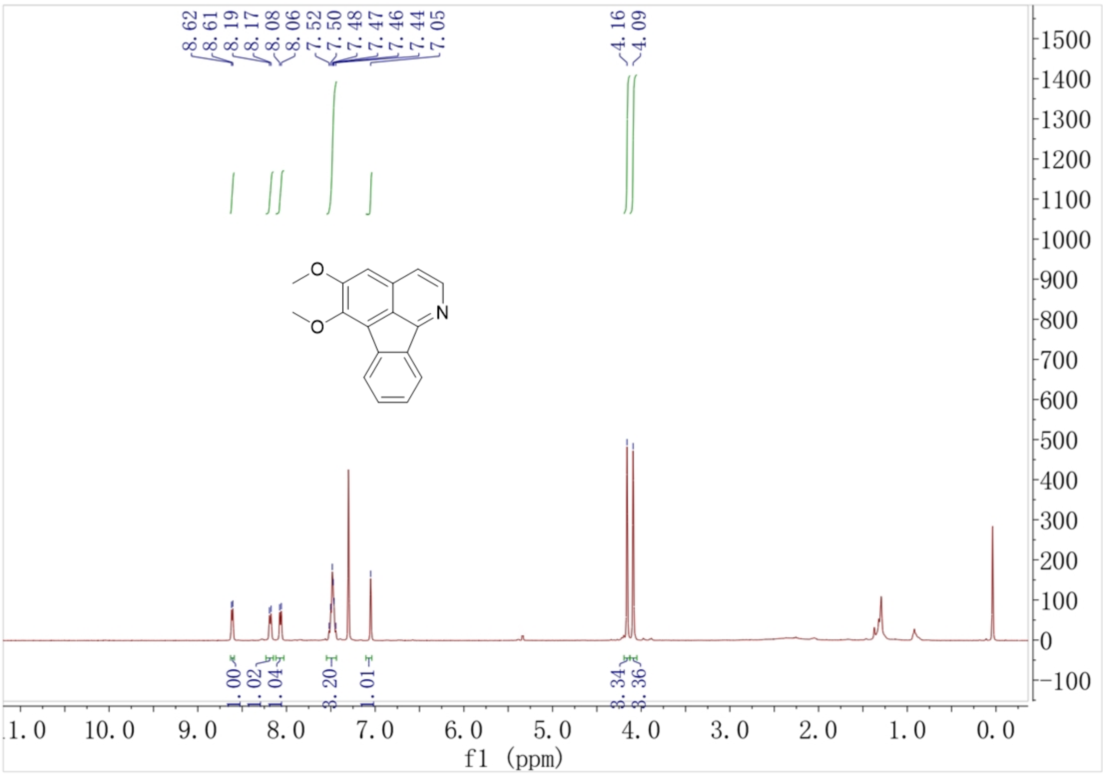

Figure S16. H and 13C NMR spectra of 2–17.

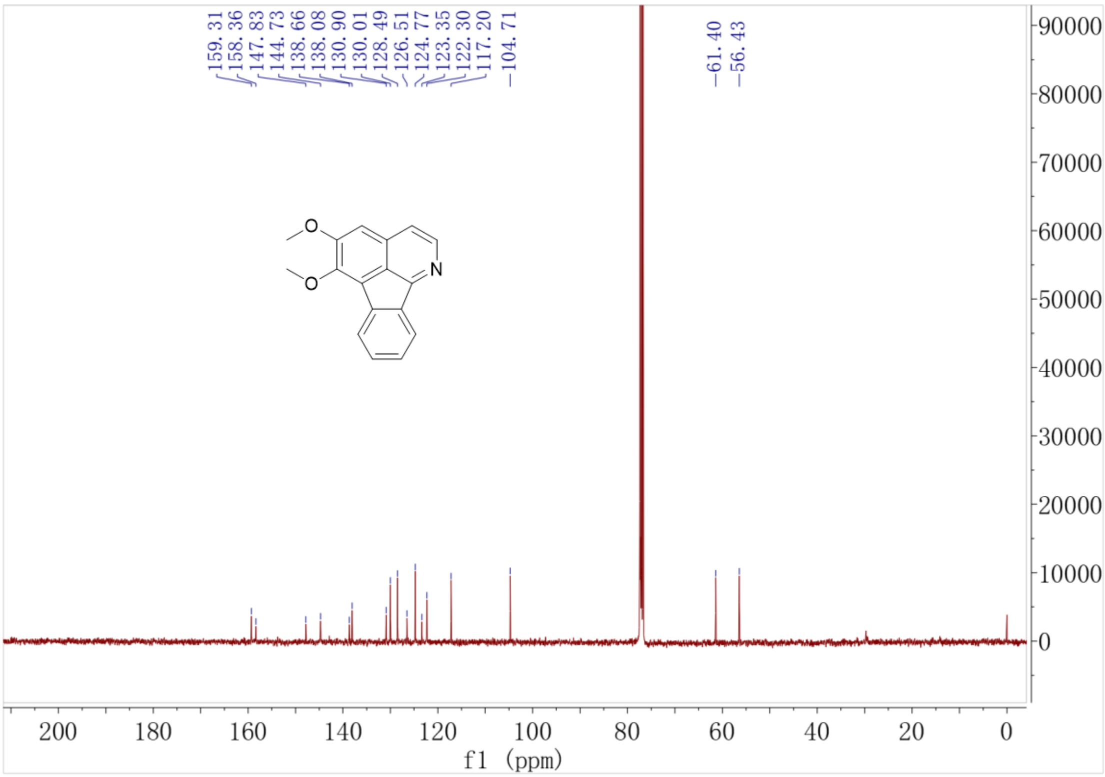

Figure S16. H and 13C NMR spectra of 2–17.
